# miR-140-3p enhanced the osteo/odontogenic differentiation of DPSCs via inhibiting KMT5B under hypoxia condition

**DOI:** 10.1038/s41368-021-00148-y

**Published:** 2021-12-07

**Authors:** Han Zheng, Ning Wang, Le Li, Lihua Ge, Haichao Jia, Zhipeng Fan

**Affiliations:** 1 Laboratory of Molecular Signaling and Stem Cells Therapy, Beijing Key Laboratory of Tooth Regeneration and Function Reconstruction, Capital Medical University School of Stomatology, Beijing, China; 2 Tsinghua University Hospital, Stomatological Disease Prevention and Control Center, Tsinghua University, Beijing, China; 3 Department of Orthodontics, Capital Medical University School of Stomatology, Beijing, China; 4 Research Unit of Tooth Development and Regeneration, Chinese Academy of Medical Sciences, Beijing, China

**Keywords:** Stem-cell differentiation, Stem-cell niche, Mesenchymal stem cells

## Abstract

Human dental pulp stem cells (DPSCs) have emerged as an important source of stem cells in the tissue engineering, and hypoxia will change various innate characteristics of DPSCs and then affect dental tissue regeneration. Nevertheless, little is known about the complicated molecular mechanisms. In this study, we aimed to investigate the influence and mechanism of miR-140-3p on DPSCs under hypoxia condition. Hypoxia was induced in DPSCs by Cobalt chloride (CoCl_2_) treatment. The osteo/dentinogenic differentiation capacity of DPSCs was assessed by alkaline phosphatase (ALP) activity, Alizarin Red S staining and main osteo/dentinogenic markers. A luciferase reporter gene assay was performed to verify the downstream target gene of miR-140-3p. This research exhibited that miR-140-3p promoted osteo/dentinogenic differentiation of DPSCs under normoxia environment. Furthermore, miR-140-3p rescued the CoCl_2_-induced decreased osteo/odontogenic differentiation potentials in DPSCs. Besides, we investigated that miR-140-3p directly targeted lysine methyltransferase 5B (KMT5B). Surprisingly, we found inhibition of KMT5B obviously enhanced osteo/dentinogenic differentiation of DPSCs both under normoxia and hypoxia conditions. In conclusion, our study revealed the role and mechanism of miR-140-3p for regulating osteo/dentinogenic differentiation of DPSCs under hypoxia, and discovered that miR-140-3p and KMT5B might be important targets for DPSC-mediated tooth or bone tissue regeneration.

## Introduction

Pulpitis and periapical periodontitis are always irreversible, and nowadays the main therapy is root canal treatment clinically, which has reported with high success rates.^1^ Except for a small part of complications of treatment, the teeth will always be more susceptible to fracture after root canal treatment resulting in a higher incidence of extraction. Mesenchymal stem cells (MSCs)-based tissue regeneration has shown great promise in modern medicine.^2–4^ MSCs from different sources have been reported and possessed special characteristics, like self-renewal and differentiation capacities, which have showed great potential for regenerating tooth and maxillofacial tissue regeneration.^5^ Today, the therapeutic effect of MSC-based tissue regeneration is not always satisfactory in diseased microenvironment.^6^ The culture of MSCs is always under normoxia in vitro. Upon administration, MSCs enter the diseased microenvironment of patients and react to the new cues which are actually inflammation and hypoxia, common to most oral disease. The hypoxia microenvironment has been uncovered to exert great influence on the MSCs functions and effects of tissue regeneration.^7^ Researchers have exhibited the different results about whether hypoxia inhibits the osteo/odontogenic differentiation of dental MSCs. It was reported that characteristics of dental MSCs changed and mineralization decreased under hypoxia.^8–10^ While other studies showed the totally opposite results in that hypoxia upregulated osteo/odontogenic-related genes in different kinds of MSCs derived from dental tissues.^11,12^ Altogether, the mechanism is still unclear and enhancing MSCs function under hypoxia environments is a key issue for dental tissue regeneration.

The extracellular environment such as a hypoxic microenvironment and intracellular changes like the expression levels of certain microRNAs (miRNAs) could additionally balance and determine MSC function and fate.^13^ MiRNAs, a sort of noncoding RNA molecules, can normally bind to the 3′-untranslated region of their target genes and then take part in the regulation of significant biological processes such as differentiation, survival, and apoptosis.^14,15^ Growing evidence suggested that under hypoxia the expression levels of some miRNAs changed. CoCl_2_-induced hypoxia upregulated the expression of hypoxia-inducible factor-1 (HIF-1) and miR-210 in bone marrow mesenchymal stem cells (BMSCs), which functioned in cell survival under hypoxia.^16^ MiR-206, a critical regulator, was decreased in BMSCs under hypoxia, which demonstrated a novel aspect of prosurvival signaling in a hypoxic microenvironment (1%O_2_).^17^ Human dental pulp stem cells (DPSCs) are seen as one of candidate stem cells for applications.^18–20^ In our previous study, we investigated the molecular mechanisms of DPSCs under hypoxia, and we systemically profiled the expression of noncoding RNAs of DPSCs in hypoxic environments, which exhibited that a part of certain miRNAs, including miR-210-3p and miR-140-3p, were differentially upregulated under hypoxia.^21^ Recently, researchers have verified that miR-140-3p can increase osteocalcin (OCN) transcription and act as a key regulatory factor between wingless/integrated (Wnt) signaling and transforming growth factor-beta (TGFβ) signaling pathways during osteoblast differentiation.^22^ Another previous study pointed miR-140-3p as potential biomarkers in relation with osteoporosis.^23^ Some study revealed that five miRNAs including miR-140-3p were highly expressed in standard healing fractures compared with unhealing fractures.^24^ Furthermore, some researchers have found that hypoxia is related with miR-140-3p, and HIF-1α is identified as a target of miR-140-3p.^25,26^ But in fact, the role and mechanism of miR-140-3p for osteo/odontogenic differentiation of DPSCs under hypoxia have been poorly understood so far.

In this research, we observed the function of miR-140-3p for osteo/odontogenic differentiation of DPSCs under hypoxia. We found miR-140-3p upregulated the osteo/dentinogenic differentiation of DPSCs by targeting KMT5B under hypoxia condition.

## Results

### MiR-140-3p was upregulated in CoCl_2_-induced hypoxia

**Fig. 1:**
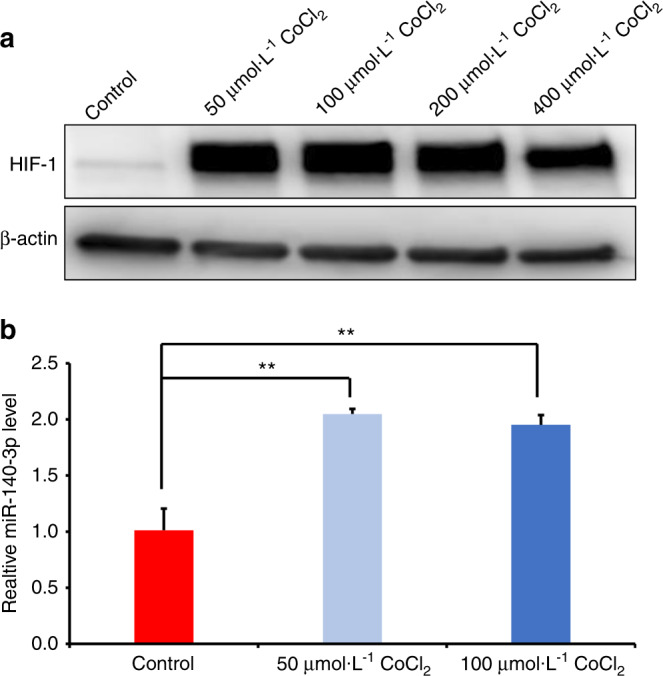
MiR-140-3p was upregulated in CoCl2-induced DPSCs. **a** Western blot results displayed the expression of HIF-1 at 48 h after different dose of CoCl_2_ treatment. **b** The expression of miR-140-3p was shown in DPSCs treated with 50, 100 μmol·L^−1^ CoCl_2_, respectively. β-actin or U6 was acted as an internal control. The one-way ANOVA was applied to confirming statistical significance. Error Bars mean standard deviations (*n* = 3). **P ≤ 0.01

The MSCs markers of DPSCs were verified, which showed that DPSCs were positive for cell surface markers CD90, CD105 and negative for CD34, CD45 (Supplementary Fig. 1). The hypoxia mimetic CoCl_2_ was used to create the hypoxia environment. Firstly, DPSCs was incubated with 0, 50, 100, 200, and 400 μmol·L^−1^ CoCl_2_ for 48 h, and HIF-1 expression was obviously enhanced in the CoCl_2_-induced group in contrast with untreated group (Fig. 1a). Then we quantified the miR-140-3p level and the result exhibited that the miR-140-3p expression in DPSCs was dramatically upregulated in CoCl_2_-induced group compared with untreated group (Fig. 1b).

### The miR-140-3p could enhance the osteo/odontogenic differentiation capacity of DPSCs

**Fig. 2:**
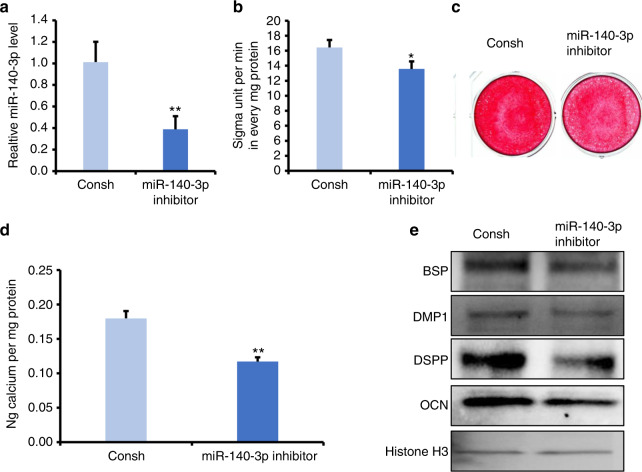
Knockdown of miR-140-3p repressed the osteo/dentinogenic differentiation of DPSCs. **a** Real-time RT-PCR exhibited the knockdown efficiency of miR-140-3p in DPSCs. **b** ALP activity assay. **c** Alizarin Red S staining. **d** Quantitative calcium measurements. **e** The OCN, DSPP, DMP1, and BSP expressions at 7 days after the induction in DPSCs. Student’s *t*-test was applied to confirming statistical significance. Histone H3 or U6 was acted as an internal control. Error Bars mean standard deviations (*n* = 3). *P ≤ 0.05; **P ≤ 0.01

To observe the role of miR-140-3p in DPSCs cultured under normoxia or hypoxia, miR-140-3p inhibitor was transfected into DPSCs. After selection for 3 days by 1 µg/ml puromycin, the knockdown efficiency was verified by real-time RT-PCR (Fig. 2a). Then, we planned to figure out whether miR-140-3p affected the osteo/odontogenic differentiation potential of DPSCs under normoxia. After 3 days of induction, the results of ALP activity assay demonstrated that knockdown of miR-140-3p decreased ALP activity (Fig. 2b). Then after 14 days of induction, in contrast with the control group, Alizarin Red S staining and quantitative calcium measurements demonstrated suppression of mineralization in miR-140-3p-knockdown DPSCs (Fig. 2c, d). Furthermore, we detected the osteo/odontogenic-related genes, such as dentin sialophosphoprotein (DSPP), bone sialoprotein (BSP), OCN, dentin matrix acidic phosphoprotein 1 (DMP1) in 7 days of induction, and Western blot results displayed that the expression of DMP1, BSP, DSPP, and OCN were downregulated in miR-140-3p-knockdown DPSCs in contrast with the control group (Fig. 2e). Moreover, CCK8 assays showed that knockdown of miR-140-3p resulted in the number of DPSCs increased at 24 h, 48 h, 72 h under normoxia. And CFSE assays revealed that knockdown of miR-140-3p increased the cell proliferation at 72 h under normoxia (Supplementary Fig. 2).

**Fig. 3:**
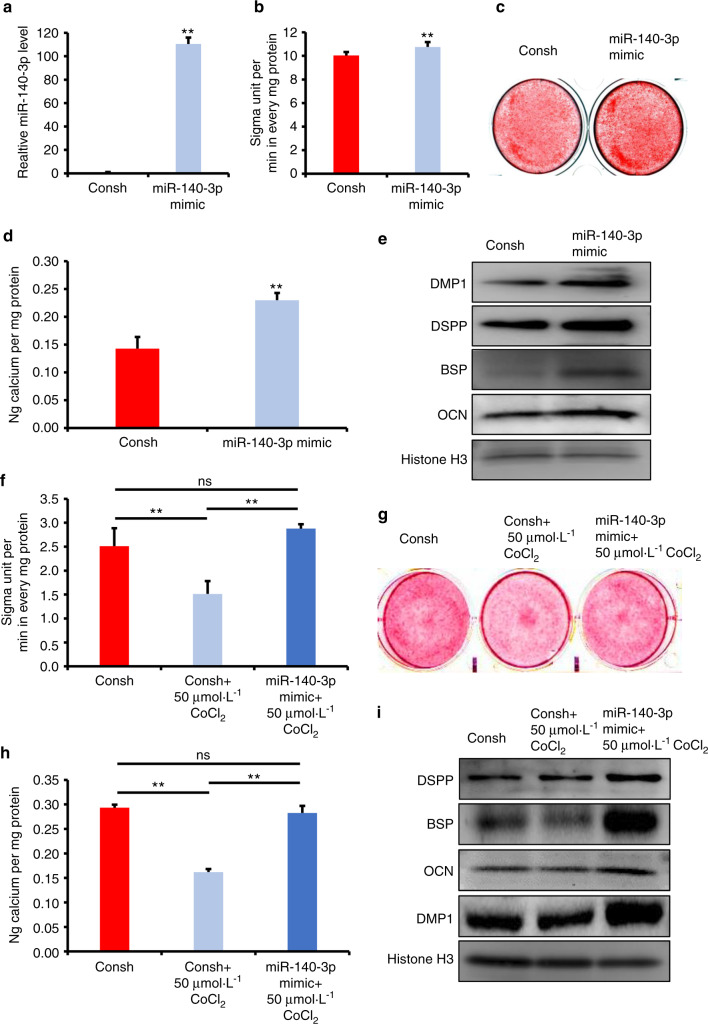
Overexpression of miR-140-3p promoted the osteo/dentinogenic differentiation of DPSCs under normoxia and hypoxia. **a** Real-time RT-PCR exhibited the overexpression efficiency of miR-140-3p in DPSCs. **b** ALP activity assay. **c** Alizarin Red S staining. **d** Quantitative calcium measurements. **e** Western blot results of the OCN, DSPP, DMP1, and BSP expressions at 7 days after the induction in DPSCs by Western blot detection. **f**–**i** 50 μmol·L^−1^ CoCl_2_ was used to treat DPSCs. **f** ALP activity assay. **g** Alizarin Red S staining. **h** Quantitative calcium measurements. **i** The OCN, DSPP, DMP1, and BSP expressions at 7 days after the induction in DPSCs by western blot detection. The one-way ANOVA or Student’s *t*-test was applied to confirming statistical significance. Histone H3 or U6 was acted as an internal control. Error Bars mean standard deviations (*n* = 3). **P ≤ 0.01

Next, we observed the function of miR-140-3p overexpression in DPSCs. DPSCs were infected with a lentiviral miR-140-3p mimic and then selected by puromycin. The overexpression efficiency was verified using real-time RT-PCR (Fig. 3a). In three days of induction, increased ALP activity was shown in miR-140-3p-overexpressing DPSCs by contrast with the control group using ALP activity assay detection (Fig. 3b). The 14 days after induction, Alizarin S Red staining combining with quantitative calcium measurements also exhibited improvement of mineralization in miR-140-3p-overexpressing DPSCs compared with the control group (Fig. 3c, d). Then in 7 days of induction, results from Western blot confirmed the increasing expression of OCN, DSPP, DMP1, and BSP in miR-140-3p-overexpressing DPSCs compared with the control group (Fig. 3e).

### The miR-140-3p rescued the hypoxia-induced reduction of osteo/odontogenic differentiation potentials in DPSCs

Next, we wondered if miR-140-3p overexpressing still had the ability to enhance the osteo/odontogenic differentiation capacity of DPSCs under hypoxic condition. Thus, 50 μmol·L^−1^ CoCl_2_ was added to the osteogenic medium to mimic hypoxia environment. Surprisingly, after 3 days of induction, ALP activity assay results demonstrated that 50 μmol·L^−1^ CoCl_2_ inhibited the ALP activity of DPSCs compared with untreated group, and miR-140-3p overexpressing enhanced the decreased ALP activity in CoCl_2_ treated DPSCs (Fig. 3f). Besides, results from Alizarin Red S staining combing with quantitative calcium measurements confirmed 50 μmol·L^−1^ CoCl_2_ suppressed the mineralization compared with untreated group, and miR-140-3p overexpressing rescued the reduced mineralization ability in CoCl_2_ treated DPSCs (Fig. 3g, h). Then using Western blot assay, we observed the protein levels of OCN, DSPP, DMP1, and BSP, which were decreased in CoCl_2_ treated DPSCs compared with untreated group, and upregulated in miR-140-overexpressing DPSCs with 50 μmol·L^−1^ CoCl_2_ stimulation (Fig. 3i). And immunocytochemistry staining also verified that the protein levels of DMP1 and DSPP were downregulated in CoCl_2_ treated DPSCs compared with untreated group, and upregulated in miR-140-overexpressing DPSCs with 50 μmol·L^−1^ CoCl_2_ stimulation (Supplementary Fig. 3). Furthermore, CCK8 assays showed that knockdown of miR-140-3p resulted in the number of DPSCs increased at 72 h under hypoxia. And CFSE assays results also showed that knockdown of miR-140-3p promoted the DPSCs proliferation at 72 h under hypoxia (Supplementary Fig. 4).

### MiR-140-3p directly inhibited KMT5B in DPSCs

**Fig. 4:**
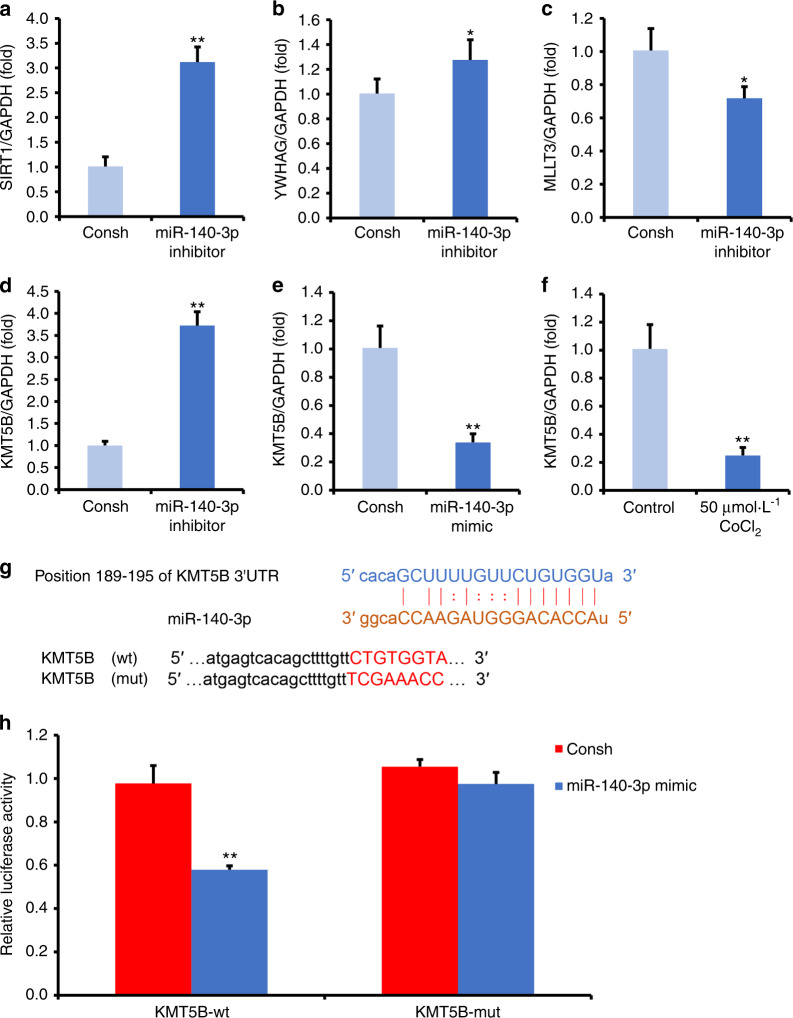
MiR-140-3p negatively regulated KMT5B in DPSCs. **a**–**d** DPSCs were transfected with a negative control (Consh) or miR-140-3p inhibitor, and SIRT1 (**a**), YWHAG (**b**), MLLT3 (**c**), KMT5B (**d**) expressions were measured by real-time RT-PCR. **e** DPSCs were transfected with a negative control (Consh) or miR-140-3p mimic, and KMT5B expression was verified by real-time RT-PCR. **f** The KMT5B expression was shown in DPSCs treated with 50 μmol·L^−1^ CoCl_2_. **g** Predicted binding site of miR-140-3p on 3′UTR of KMT5B. **h** Dual-luciferase reporter assays. Student’s *t*-test was applied to confirming statistical significance. GAPDH was acted as an internal control. Error Bars mean standard deviations (*n* = 3). *P ≤ 0.05; **P ≤ 0.01

To better understand the mechanism of miR-140-3p in DPSCs, then the potential targets of miR-140-3p were predicted using online target gene prediction software (miRmap, TargetScan, PITA, and PicTar online). Sirtuin-1 (SIRT1), tyrosine 3-monooxygenase/tryptophan 5-monooxygenase activation protein gamma (YWHAG), mixed‐lineage leukemia translocated to chromosome 3 protein (MLLT3), and lysine methyltransferase 5B (KMT5B) were predicted as the potential target of miR-140-3p. By real-time RT-PCR, we found that SIRT1, YWHAG, and KMT5B were upregulated, and MLLT3 was downregulated in miR-140-3p-knowkdown DPSCs in contrast with the control group (Fig. 4a, b, c, d). Next, we chose one candidate target, KMT5B, to do further investigation in DPSCs. In miR-140-3p-overexpressing DPSCs, we confirmed the expression of KMT5B was decreased (Fig. 4e). Interestingly, the expression of KMT5B was also downregulated in 50 μmol·L^−1^ CoCl_2_ treated DPSCs compared with untreated group (Fig. 4f). Then, by online prediction software, potential binding sites of miR-140-3p in the 3′UTR of KMT5B were identified (Fig. 4g). Next, we used Dual-luciferase reporter assays to verify whether KMT5B was a direct target gene of miR-140-3p. The results showed that the luciferase activity was decreased in 293 T cells which double transfected with miR-140-3p mimic and the wild-type KMT5B 3′UTR reporter group in contrast with the control group, while the luciferase activity was no difference in 293 T cells which double transfected with miR-140-3p mimic and the mutant KMT5B 3′UTR reporter group compared with the control group (Fig. 4h).

### KMT5B significantly suppressed the osteo/odontogenic differentiation of DPSCs

**Fig. 5:**
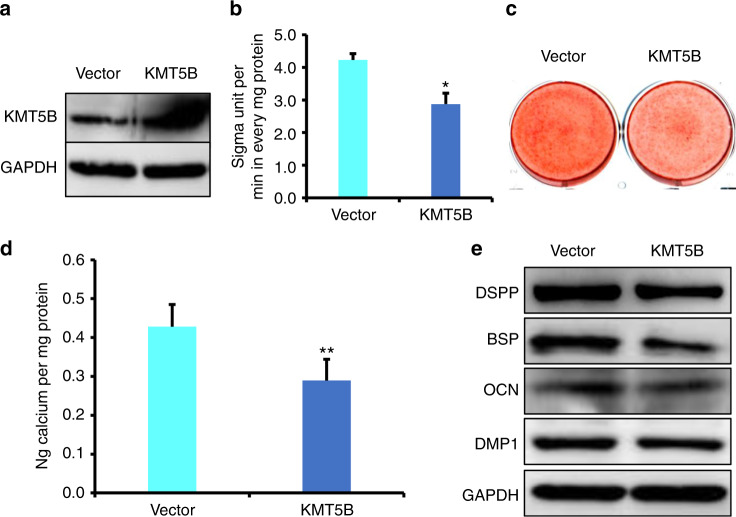
Overexpression of KMT5B repressed the osteo/dentinogenic differentiation of DPSCs. **a** Western blot exhibited the overexpression of KMT5B in DPSCs. **b** ALP activity assay. **c** Alizarin Red S staining. **d** Quantitative calcium measurements. **e** The OCN, DSPP, DMP1, and BSP expressions at 7 days after the induction in DPSCs by western blot detection. Student’s *t*-test was applied to confirming statistical significance. GAPDH was acted as an internal control. Error Bars mean standard deviations (*n* = 3). *P ≤ 0.05; **P ≤ 0.01

**Fig. 6:**
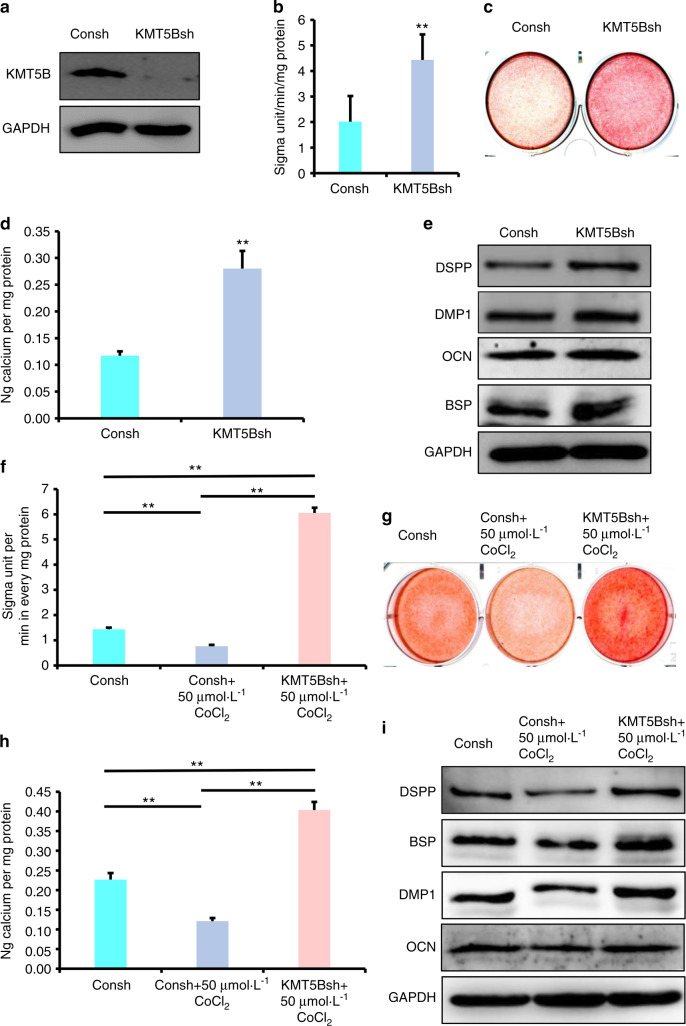
The depletion of KMT5B enhanced the osteo/dentinogenic differentiation of DPSCs under normoxia and hypoxia. **a** The knockdown efficiency of KMT5B by western blot detection. **b** ALP activity assay. **c** Alizarin Red S staining. **d** Quantitative calcium measurements. **e** The OCN, DSPP, DMP1, and BSP expressions at 7 days after the induction in DPSCs by western blot detection. **f**–**i** 50 μmol·L^−1^ CoCl_2_ was used to treat DPSCs. **f** ALP activity assay. **g** Alizarin Red S staining. **h** Quantitative calcium measurements. **i** The OCN, DSPP, DMP1, and BSP expressions at 7 days after the induction in DPSCs by western blot detection. The one-way ANOVA or Student’s *t*-test was applied to confirming statistical significance. GAPDH was acted as an internal control. Error Bars mean standard deviations (*n* = 3). **P ≤ 0.01

Furthermore, the function of KMT5B was investigated in DPSCs. Wild-type KMT5B construct was transfected into DPSCs. We verified the overexpression efficiency of KMT5B by Western blot (Fig. 5a). Three days after the induction, ALP activity assay results confirmed that overexpression of KMT5B inhibited ALP activity in comparison with the control group (Fig. 5b). After 14 days of induction, Alizarin Red S staining and quantitative calcium measurements demonstrated that mineralization was also inhibited in KMT5B overexpressing DPSCs in contrast with the control group (Fig. 5c, d). Western blot results displayed that the expression of BSP, DSPP, DMP1, and OCN were decreased in KMT5B overexpressing DPSCs at 7 days after the induction (Fig. 5e). In addition, DPSCs were transfected with KMT5B shRNA to knock down the endogenous expression of KMT5B. The knockdown efficiency of KMT5B in DPSCs was proved to be meaningful by Western blot (Fig. 6a). Three days after osteogenic induction, we discovered that the knockdown of KMT5B obviously enhanced ALP activity compared with the control group (Fig. 6b). Then, as assessed by Alizarin Red S staining along with quantitative calcium measurements, knockdown of KMT5B remarkably promoted mineralization in contrast with the control group (Fig. 6c, d). In addition, assayed by Western blot, knockdown of KMT5B significantly upregulated the expression of DMP1, OCN, DSPP, and BSP in 7 days of induction (Fig. 6e).

### The depletion of KMT5B rescued the hypoxia-induced reduction of osteo/odontogenic differentiation potentials in DPSCs

Next, we wondered if KMT5B depletion had the ability to enhance the osteo/odontogenic differentiation capability of DPSCs under hypoxia. 50 μmol·L^−1^ CoCl_2_ was added to the osteogenic medium to mimic hypoxia environment. Three days after osteogenic induction, the depletion of KMT5B enhanced the decreased ALP activity in CoCl_2_ treated DPSCs compared with vector group (Fig. 6f). Alizarin Red S staining and quantitative calcium measurements results showed that the depletion of KMT5B rescued the reduced mineralization ability in CoCl_2_ treated DPSCs compared with vector group (Fig. 6g, h). Then Western blot results verified that OCN, DSPP, DMP1, and BSP were downregulated in CoCl_2_ treated DPSCs compared with untreated group, and upregulated in KMT5B depletion DPSCs with 50 μmol·L^−1^ CoCl_2_ stimulation (Fig. 6i).

## Discussion

The hypoxia microenvironment has been reported to affect the function and the curative effect of MSCs, and we aim to investigate the molecular mechanism of MSCs under hypoxia to find potential target for improving MSCs function.^27–29^ Emerging evidence showed that miRNAs were widely involved in a variety of biological functions such as proliferation, apoptosis and differentiation of MSCs under the condition of hypoxia.^30–33^ Our previous result displayed that miR-140-3p was upregulated in DPSCs under 3% hypoxia.^21^ And in this research, we further investigated the role of miR-140-3p in DPSCs under hypoxia. Cobalt chloride (CoCl_2_) has been seen as a useful hypoxia mimetic, inhibiting the formation of oxygenated hemoglobin.^34,35^ During cell culture, CoCl_2_ repressed the catalysis of prolyl hydroxylases, creating an intracellular hypoxia-like state.^36^ Hence, CoCl_2_ was applied in this research to mimic hypoxia. CoCl_2_ has been seen as a hypoxia-mimicking agent to create the hypoxia condition via inducing the accumulation of hypoxia-inducible factor-1 (HIF-1), an oxygen-sensitive transcriptional activator.^37–39^ The HIF-1 is a critical oxygen-sensitive transcriptional activator, which is hydroxylated and becomes proteasomal degradation at higher oxygen tensions, but under hypoxia, HIF-1 becomes more stable, and serves as transcription factors.^40–42^ In the present research, we detected the influence of CoCl_2_ on HIF-1 expression at different dose, and found that 50 μmol·L^−1^, 100 μmol·L^−1^, 200 μmol·L^−1^, and 400 μmol·L^−1^ CoCl_2_ had a similar effect on HIF-1 expression. Then, we used 50 μmol·L^−1^ and 100 μmol·L^−1^ CoCl_2_ to treat DPSCs, and verified that miR-140-3p expression was upregulated as same level in 50 μmol·L^−1^ and 100 μmol·L^−1^ CoCl_2_ treated DPSCs, which meant CoCl_2_-induced hypoxia increased miR-140-3p expression, which was consistent with our previous study. Considering the toxicity of CoCl_2_, we chose 50 μmol·L^−1^ CoCl_2_ to mimic hypoxia in the following experiment, and we found that 50 μmol·L^−1^ CoCl_2_ repressed the differentiation capacity of DPSCs by detecting ALP activity, mineralization in vitro, and the indicators of osteo/odontogenic differentiation including DMP1, OCN, DSPP, and BSP, which was supported by our previous research that hypoxia (3%O_2_) repressed the osteo/odontogenic differentiation capacity of DPSCs.^21^

Then, we performed gain- and loss- of- function assays to test the function of miR-140-3p. Results from ALP activity, mineralization in vitro, and the expression of DMP1, OCN, BSP, and DSPP comprehensively clarified the knockdown of miR-140-3p restrained the osteo/dentinogenic differentiation of DPSCs. Accordingly, the overexpression of miR-140-3p facilitated osteo/dentinogenic differentiation under normoxia. Furthermore, we found that the overexpression of miR-140-3p reversed the suppression in osteo/odontogenic differentiation of DPSCs caused by CoCl_2_-induced hypoxia. There were lots of factors that could affect differentiation of DPSCs under hypoxia, some genes play positive effect, some genes play negative effect. But totally effect of genes caused osteo/odontogenic differentiation of DPSCs decreased under hypoxic conditions. In present study, it was found that miR-140-3p enhanced the osteo/odontogenic differentiation of DPSCs, and hypoxia environment upregulated the expression of miR-140-3p in DPSCs. It indicated that hypoxia environment upregulated the expression of miR-140-3p, but the upregulated endogenous miR-140-3p could not completely antagonize the negative effect of other genes, so the phenotype in DPSCs was still decreased osteo/odontogenic differentiation. And these suggested that we need more miR-140-3p to antagonize the negative effect in hypoxia environment. Indeed, when we over-expressed exogenous miR-140-3p in DPSCs, they could completely antagonize the negative effect of other genes, and the phenotype in DPSCs became enhanced osteo/odontogenic differentiation. These results verified overexpression of miR-140-3p could enhance the osteo/odontogenic differentiation of DPSCs under hypoxia condition, which indicated that miR-140-3p might have a meaningful effect on hypoxia, suggesting miR-140-3p as a novel target for dental tissue regeneration.

Furthermore, we observed the mechanism of miR-140-3p in DPSCs. MiRNAs worked post-transcriptionally by mainly base-pairing to the 3ʹ-untranslated regions of targets genes.^43^ Therefore, a promising target of miR-140-3p is needed for further investigating in DPSCs. Researchers have verified some targets of miR-140-3p. Some studies reported that miR-140-3p was involved in osteoblast differentiation as a critical regulatory factor, and demonstrated *TGF*β*3* acted as a direct target of miR-140-3p.^22^ Moreover, miR-140-3p had inhibitory effect on preosteoblast viability and differentiation via targeting MCF2L in osteoporosis.^44^ In the stem cell differentiation, some reported that miR-140-3p negatively regulated osteogenic differentiation of BMSCs, which could be reversed by Spred2.^45^ Besides, miR-140 targeted RALA and regulated chondrogenic differentiation of MSCs by translational enhancement of SOX9 and ACAN.^46^ In present study, we used the TargetScan, miRmap, PITA, and PicTar software to predict the potential targets of miR-140-3p. SIRT1, YWHAG, MLLT3, and KMT5B were predicted as the potential target of miR-140-3p. Further we found that knockdown of miR-140-3p upregulated SIRT1, YWHAG, and KMT5B, while downregulated MLLT3 at mRNA level in DPSCs. It was reported that SIRT1, a NAD-dependent histone deacetylase, played a positive role in osteogenic differentiation of DPSCs treated by TNF-α.^47^ In the process of serial subculture during osteogenic differentiation of MSCs, some researchers found a number of differentially regulated proteins. It was showed that 14-3-3 protein gamma (YWHAG) was upregulated during the later passage, which provided some clues for regulating the osteogenic differentiation process.^48^ Histone modifications including methylation of key lysine residues play a crucial role in lots of cellular events.^49^ KMT5B encodes a protein containing a SET domain, belonging to one group of histone methyltransferases (KMTs). Although the function of KMT5B in stem cells was little known, it has been reported that certain genes of the KMT family could regulate the osteo/odontogenic differentiation of dental tissue derived MSCs.^50,51^ Enhancer of zeste homolog 2 (EZH2), also known as KMT6A, was one of widely studied SET domain-containing KMTs concerning MSC differentiation.^52^ EZH2 was a methyltransferase for H3 lysine 27 trimethylation, which could suppress osteogenic differentiation of DPSCs while promoting proliferation, indicating its role in dental pulp regeneration.^53^ Thus, we selected KMT5B for further study. And we found the expression of KMT5B was reversed by miR-140-3p and reversely correlated with miR-140-3p in CoCl_2_-induced hypoxia, which indicated that KMT5B might be the key target of miR-140-3p under hypoxia. Furthermore, using dual-luciferase reporter assay, we verified miR-140-3p binding into the binding element of the KMT5B 3ʹUTR region. These results demonstrated miR-140-3p directly targeted KMT5B in DPSCs.

Then we wondered whether miR-140-3p affected the osteo/odontogenic differentiation via KMT5B, and investigated the function of KMT5B in DPSCs. The gain- and loss- of- function assay results exhibited that KMT5B repressed the osteo/odontogenic differentiation of DPSCs showing by ALP activity, mineralization in vitro, and the protein levels of DMP1, BSP, OCN, and DSPP. Interestingly, the knockdown of KMT5B remarkably enhanced the hypoxia-impaired differentiation capacity of DPSCs. Taken together, these evidences indicated that miR-140-3p could increase the impaired osteo/odontogenic differentiation capacity of DPSCs under hypoxic conditions via directly targeting KMT5B.

## Conclusion

To sum up, in present study, the miR-140-3p could restore impaired osteo/odontogenic differentiation potentials of DPSCs under hypoxic conditions. Moreover, this study explored that miR-140-3p directly targeted KMT5B, and the depletion of KMT5B could remarkably rescue the impaired osteo/odontogenic differentiation of DPSCs under hypoxic conditions. These suggested that miR-140-3p enhanced the hypoxia-impaired osteo/odontogenic differentiation potentials of DPSCs via negatively regulating KMT5B, revealed a new mechanism for regulating osteo/dentinogenic differentiation of DPSCs in a hypoxic microenvironment, and provided the potential targets miR-140-3p and KMT5B for DPSC-mediated tissue regeneration.

## Materials and methods

### DPSC culture and drug administration

Dental pulps were physically obtained from the crown and root and then subjected to a collagenase and dispase digest. Simply, we acquired, cultured, and then identified DPSCs as previously stated.^54^ DPSCs from young group at passages 3–5 were used in this study.

To mimic the hypoxia condition, CoCl_2_ was diluted to 50 μmol·L^−1^, 100 μmol·L^−1^, 200 μmol·L^−1^, 400 μmol·L^−1^ for actual use. In order to induce osteo/odontogenic differentiation, DPSCs were plated at a density of 2 × 10^5^ cells per plate in six-well plates. Then DPSCs were changed into the differentiation medium containing β-glycerophosphate, dexamethasone and inorganic phosphate after cells reached 80% confluence.

### Viral infection

MiR-140-3p inhibitor, miR-140-3p mimic, KMT5B shRNA and control shRNA (Consh) lentivirus were all designed and synthesized by GenePharma (Suzhou, China). The miR-140-3p inhibitor sequence was 5′-CCGTGGTTCTACCCTGTGGTA-3′, the miR-140-3p mimic sequence was 5′-TACCACAGGGTAGAACCACGG-3′, KMT5B shRNA (KMT5Bsh) sequence was 5′-GGAGAAATGGAGGCAAGTTGT-3′ and the control shRNA sequence was 5ʹ-TTCTCCGAACGTGTCACGT-3ʹ. Besides, KMT5B and Vector were also purchased from GenePharma. Abiding by the manufacturer’s protocol, DPSCs transfected with lentiviruses lasted 12 h along with 6 mg/ml polybrene. Then infected cells were selected by 1 µg·mL^−1^ puromycin.

### Alkaline phosphatase (ALP) activity and Alizarin Red S staining

About the ALP activity assay, DPSCs were seeded in six-well plates and changed to osteogenic differentiation medium for 3 days. Next, in order to confirm mineralization, DPSCs were induced for 14 days, and we used 70% ethanol to fix these cells. 2% Alizarin red was then applied to staining the formation of mineralized nodules, as previously stated.^55^

### RNA extraction and real-time RT-PCR

Trizol reagent (Invitrogen) was used to extract total RNA from DPSCs. The levels of miR-140-3p were observed as previously stated.^56^ Besides, following the manufacturer’s protocol (Invitrogen), 2 μg RNA sample was reverse transcribed into cDNA. The KMT5B primers are as follows: forward, 5′-GAGAAATGGAGGCAAGTTGTCT-3′; and reverse, 5′-ACATAGCGACTCTGTCCTTCA-3′. The SIRT1 primers are as follows: forward, 5′-TAGCCTTGTCAGATAAGGAAGGA-3′; and reverse, 5′-ACAGCTTCACAGTCAACTTTGT-3′. The YWHAG primers are as follows: forward, 5′-AGCCACTGTCGAATGAGGAAC-3′; and reverse, 5′-CTGCTCAATGCTACTGATGACC-3′. The MLLT3 primers are as follows: forward, 5′-TTTGTGGAGAAAGTCGTCTTCC-3′; and reverse, 5′-GAGGTGATTCACTGGTGGATG-3′. The GAPDH primers are as follows: forward, 5′-CGGACCAATACGACCAAATCCG-3′; reverse, 5′-AGCCACATCGCTCAGACACC-3′.

### Western blot

Total proteins were extracted from DPSCs using Radio immunoprecipitation assay (RIPA) lysis buffer and protease inhibitors. 25 μg protein from each sample was prepared for Western blot analysis, which was performed as previously stated.^57^ The primary antibodies used in this study are as follows: DMP1 (Cat No. bs12359R, Bioss), HIF-1 (Cat No. 36169, Cell Signaling Technology), Histone H3 (Cat No.17168-1-AP, proteintech), OCN (Cat No. bs4917R, Bioss), KMT5B (Cat No. ab118659, abcam), DSPP (Cat No. bs10316R, Bioss), GAPDH (Cat No. 60004-1-Ig, proteintech), β-actin (Cat No. C1313; Applygen, China), BSP (Cat No. bs2668R, Bioss).

### Dual-luciferase reporter assays

Before transfection, 293 T cells (1.0 × 10^5^ cells per well) were seeded in 12-well plates and incubated overnight. MiR-140-3p mimic or mimic control along with the wild-type (wt) or mutant (mut) KMT5B 3′UTR-Luc reporter construct was then cotransfected into 293 T cells, as previously stated.^56^ 48 h later, luciferase enzymatic activities were then detected using the Dual-Luciferase Reporter Assay Kit (Vazyme).

### Cell Counting Kit-8 (CCK8) Assay

CCK8 assay (MCE, Cat. HY-K0301) was used to observe cell proliferation. DPSCs (5 × 10^3^ cells per well) were seeded into 96-well plates. At 24 h, 48 h or 72 h, the culture medium was removed and changed into 100 µL MEM mixed with 10 µL CCK8. And the plate was incubated for 1 h at 37 °C. A multiwell spectrophotometer was used to detected absorbance (OD) at 450 nm.

### Carboxyfluorescein succinimidyl ester (CFSE) assay

In short, DPSCs were labelled as previously depicted.^56^ A flow cytometry was then used to detect the labeling cells 3 days later. The data was analyzed using MODFIT LT.

### Immunocytochemical staining

The DPSCs were seeded on coverslips in 24-well plates. 24 h later, the DPSCs were changed into the differentiation medium and meanwhile 50 μmol·L^−1^ CoCl_2_ was added into CoCl_2_ treated group. After 7 days osteo/odontogenic induction, the DPSCs were fixed for about 30 min. Simply, after incubated in 3% H_2_O_2_ for 10 min followed by three PBS washes, goat serum was applied. Importantly, the primary antibodies were as follows: DSPP polyclonal antibody (1:100), DMP1 polyclonal antibody (1:100). Finally, the cells were counterstained with haematoxylin and observed under a microscope.

### Statistics analysis

SPSS 10 software was used to analyze data. One-way ANOVA or student’s *t*-test was used to assess the statistical differences, with a *P*-value ≤ 0.05 deemed to be significant.

## Supplementary information

supplementary information

western gels
