# Supplementary material for: miR-140-3p enhanced the osteo/odontogenic differentiation of DPSCs via inhibiting KMT5B under hypoxia condition
Source: Int J Oral Sci. 2021 Dec 7;13:41. doi: 10.1038/s41368-021-00148-y (PMC8651682; doi:10.1038/s41368-021-00148-y)
Supplement: Supplementary file 1 — supplementary information [file 41368_2021_148_MOESM1_ESM.docx]

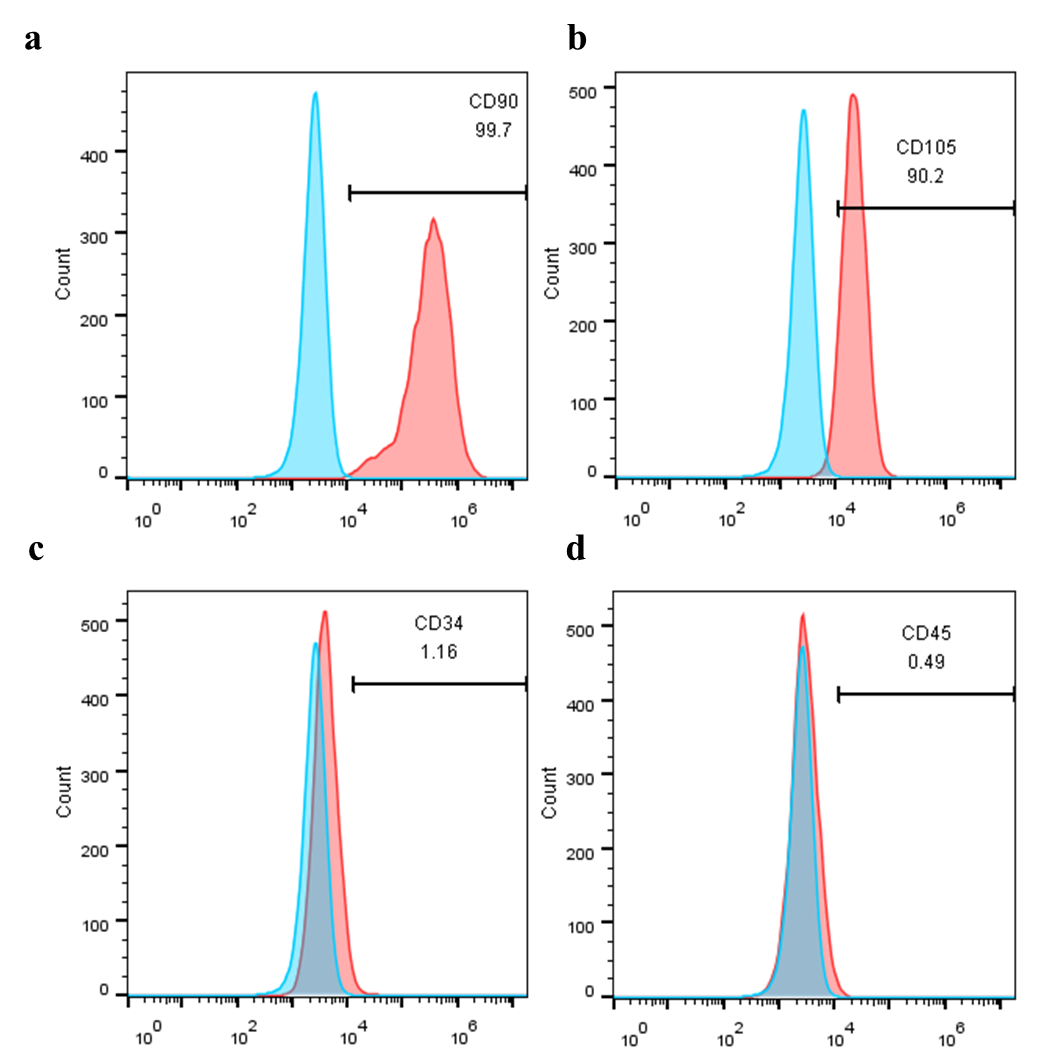


**SUPPLEMENTARY FIGURE 1.** Flow cytometric analysis of MSC markers in DPSCs. (A) CD90 (99.7%), (B) CD105 (90.2%), (C) CD34 (1.16%), (D) CD45 (0.49%).


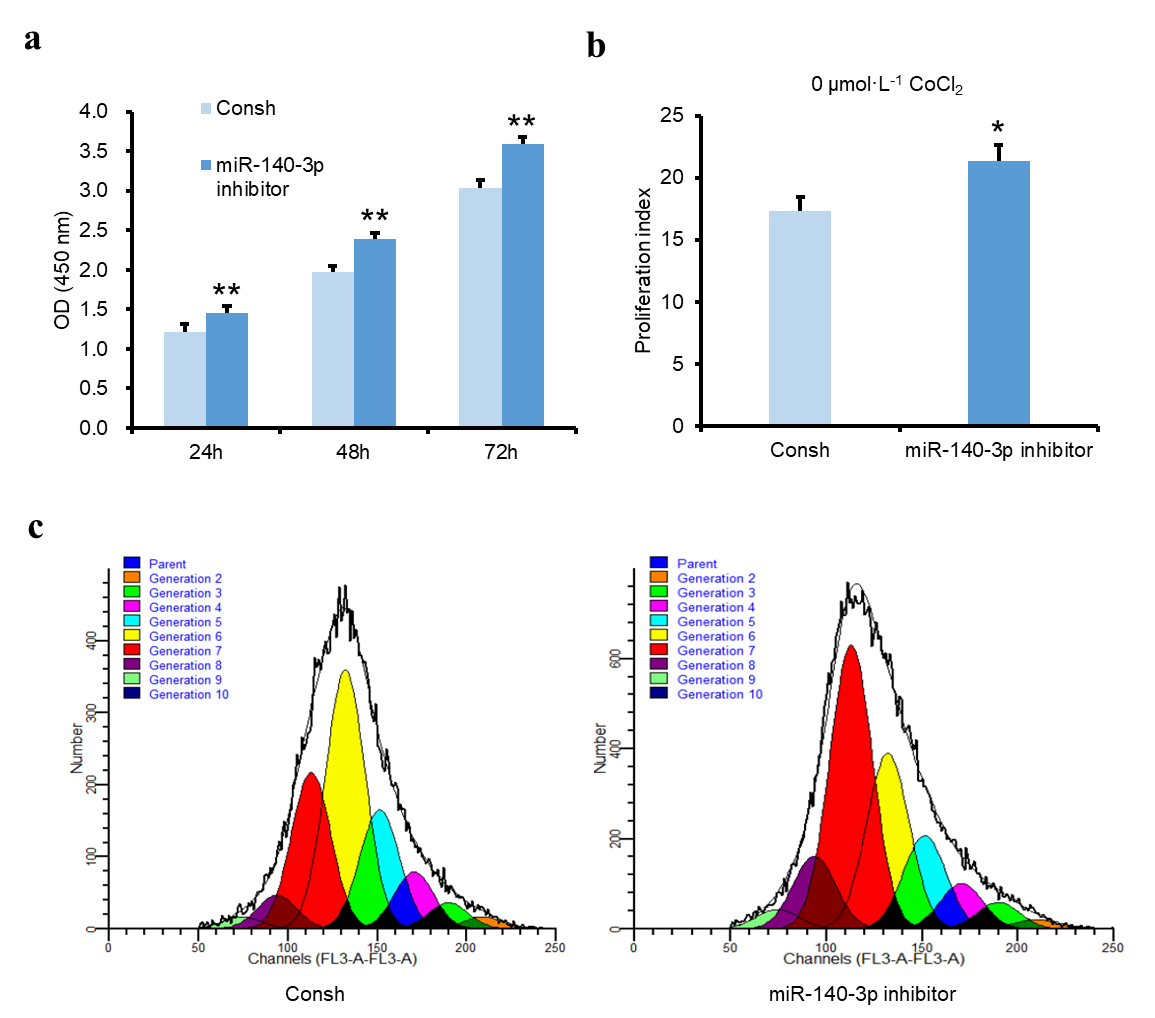


**SUPPLEMENTARY FIGURE 2.** **Knock-down of miR-140-3p promoted cell proliferation in DPSCs under normoxia.** (A) CCK8 assays results in miR-140-3p knock-down DPSCs under normoxia. (B, C) CFSE assays results in miR-140-3p knock-down DPSCs under normoxia. Student's t test was used to determine statistical significance. Error Bars represent standard deviations (n=3). *P ≤ .05; **P ≤ .01.


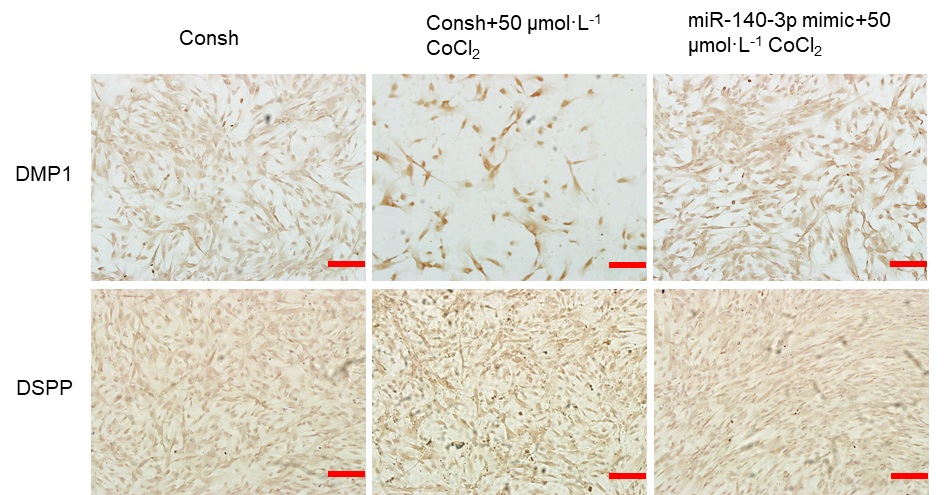


**SUPPLEMENTARY FIGURE 3.** Immunocytochemical staining results of DSPP, DMP1. Scale bar: 200 μm


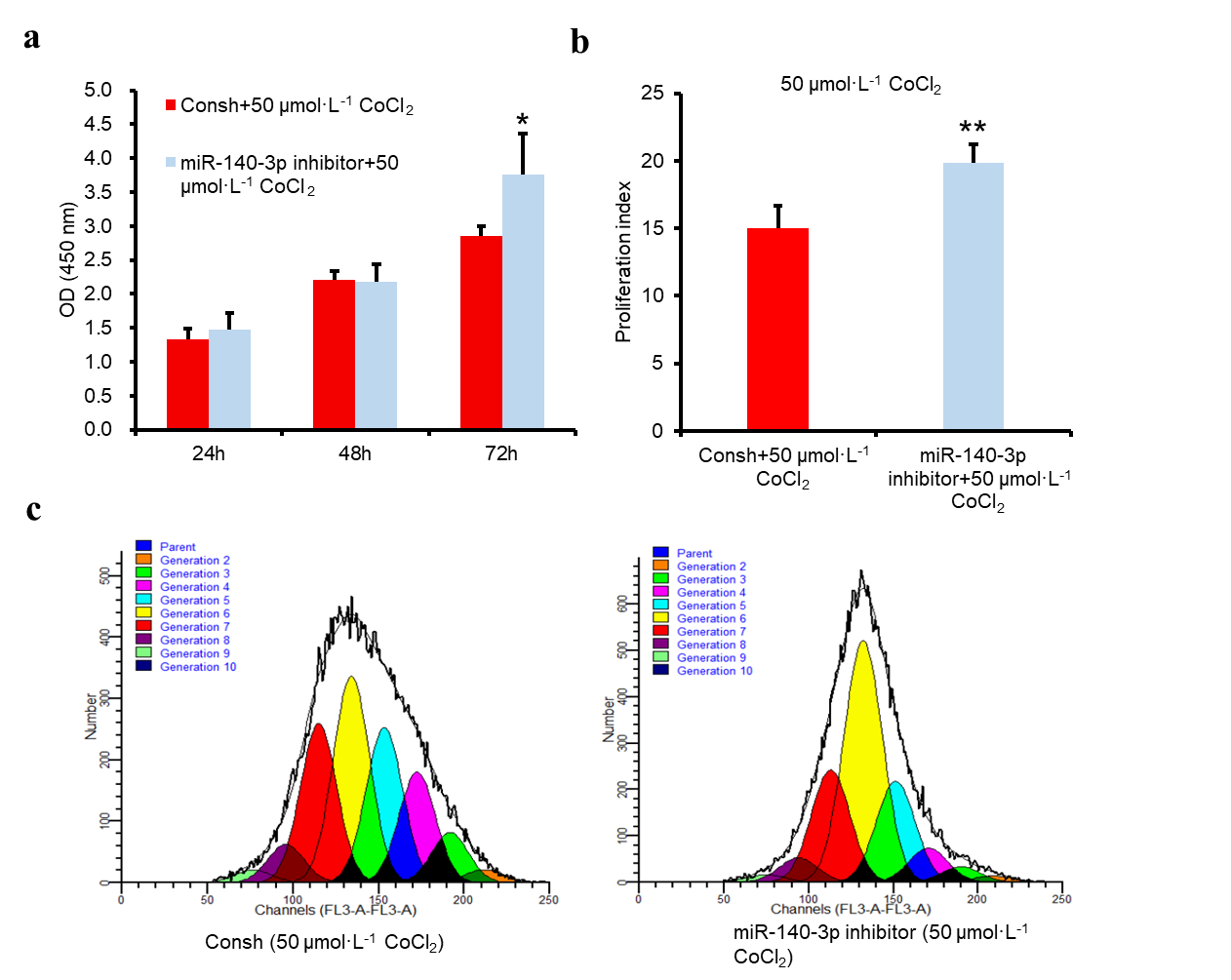


**SUPPLEMENTARY FIGURE 4.** **Knock-down of miR-140-3p promoted cell proliferation in DPSCs under hypoxia.** (A) CCK8 assays results in miR-140-3p knock-down DPSCs under hypoxia. (B, C) CFSE assays results in miR-140-3p knock-down DPSCs under hypoxia. Student's t test was used to determine statistical significance. Error Bars represent standard deviations (n=3). *P ≤ .05; **P ≤ .01.
