# Supplementary material for: miR-140-3p enhanced the osteo/odontogenic differentiation of DPSCs via inhibiting KMT5B under hypoxia condition
Source: Int J Oral Sci. 2021 Dec 7;13:41. doi: 10.1038/s41368-021-00148-y (PMC8651682; doi:10.1038/s41368-021-00148-y)

## Fig.1a

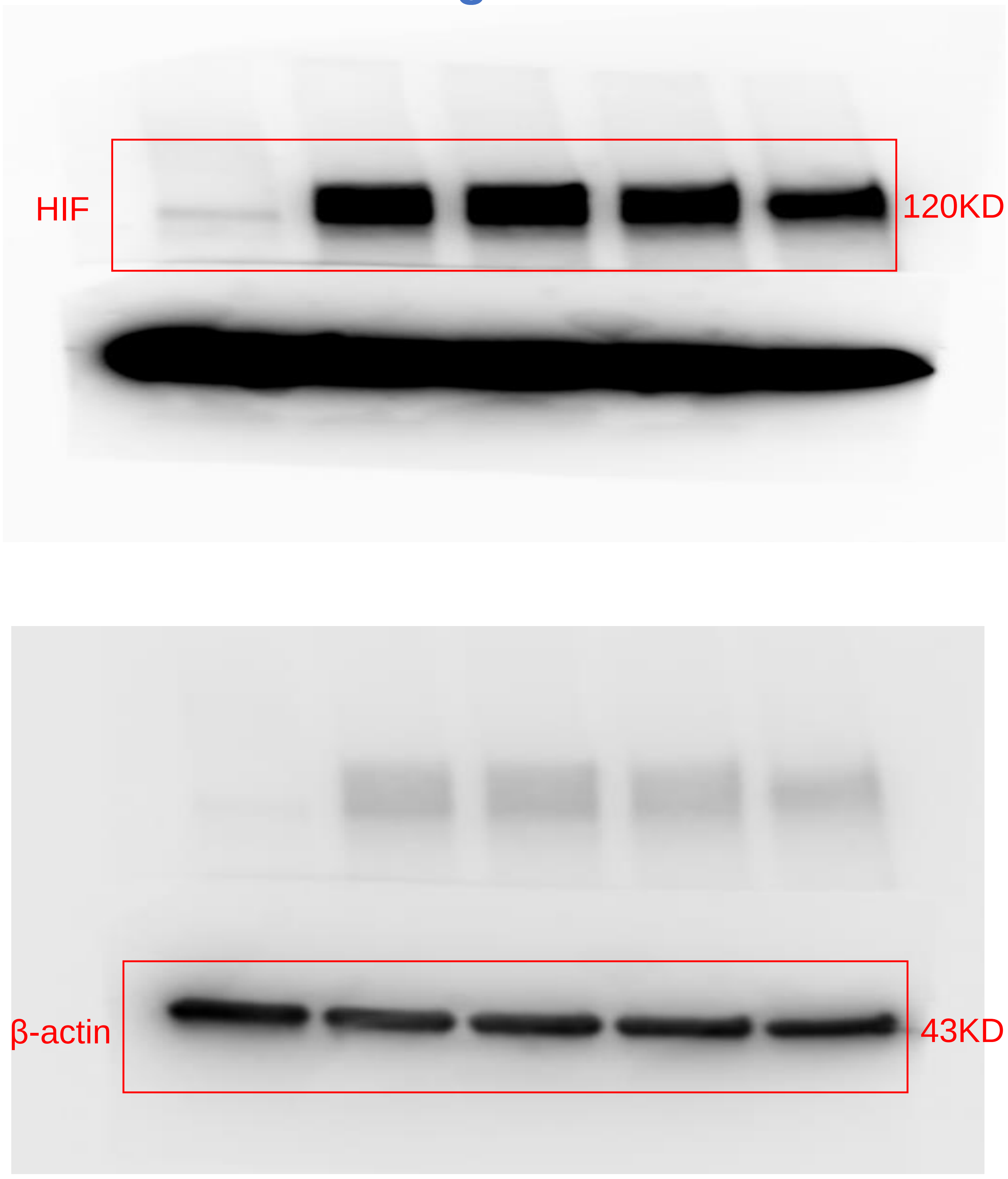

# Fig.2e

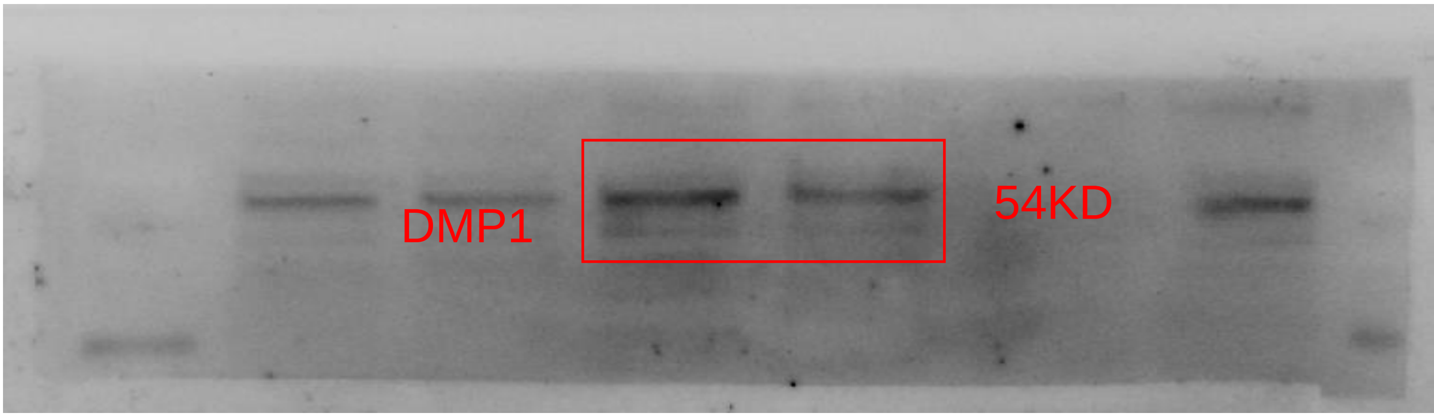


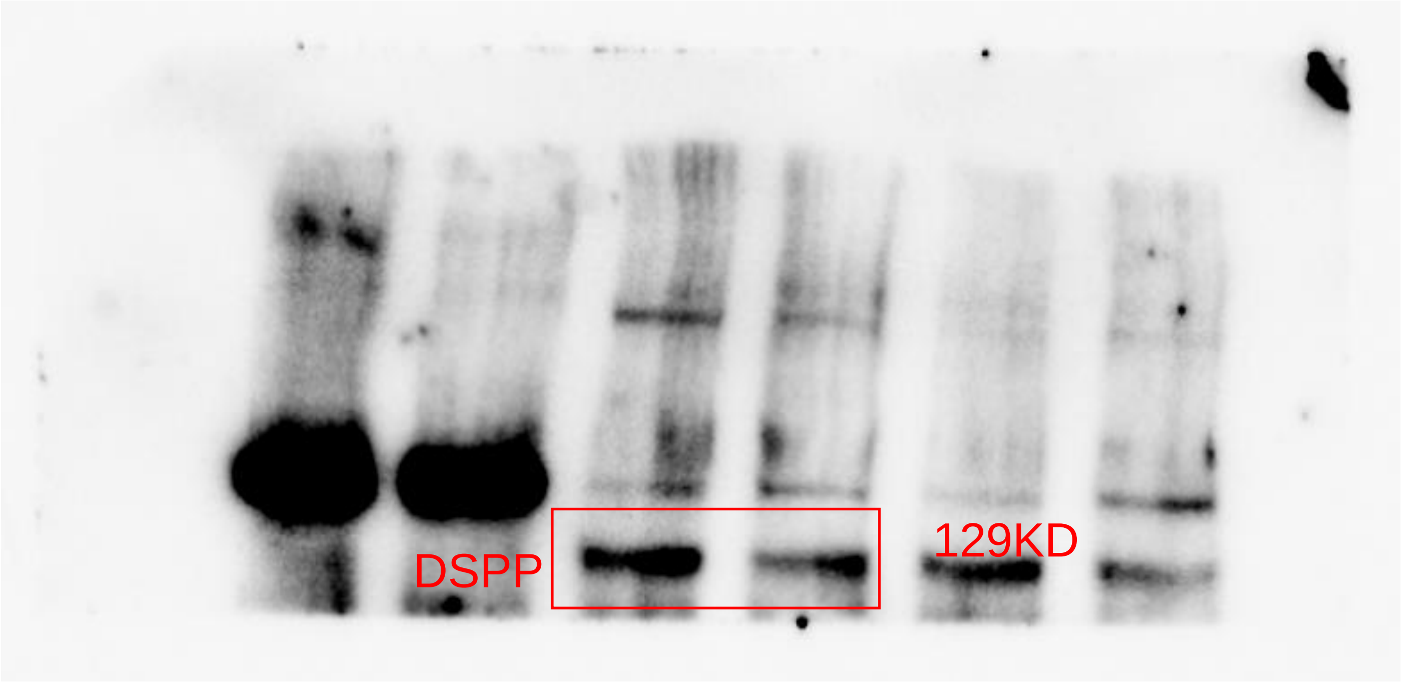

Fig.2e

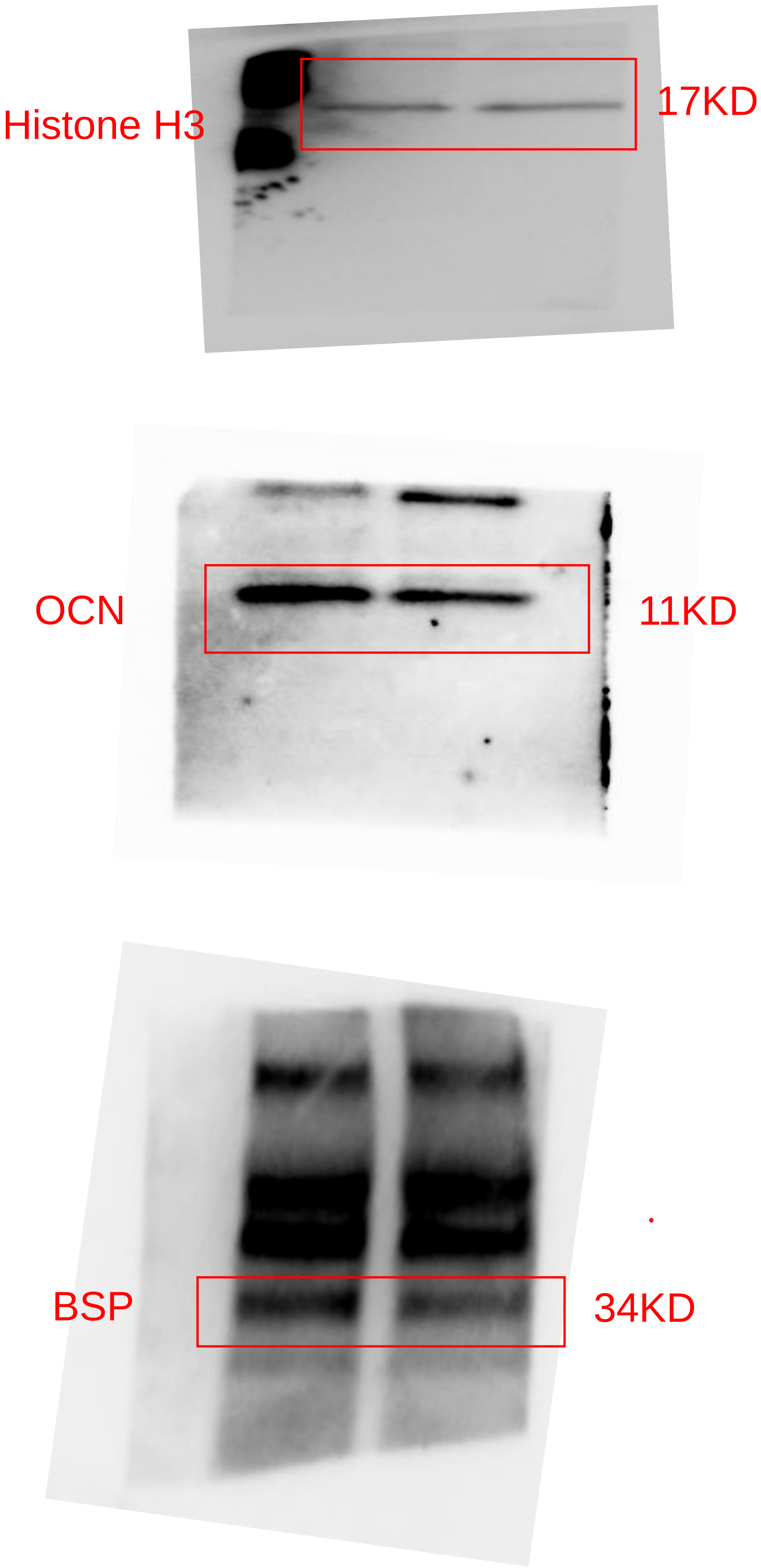

# Fig.3e

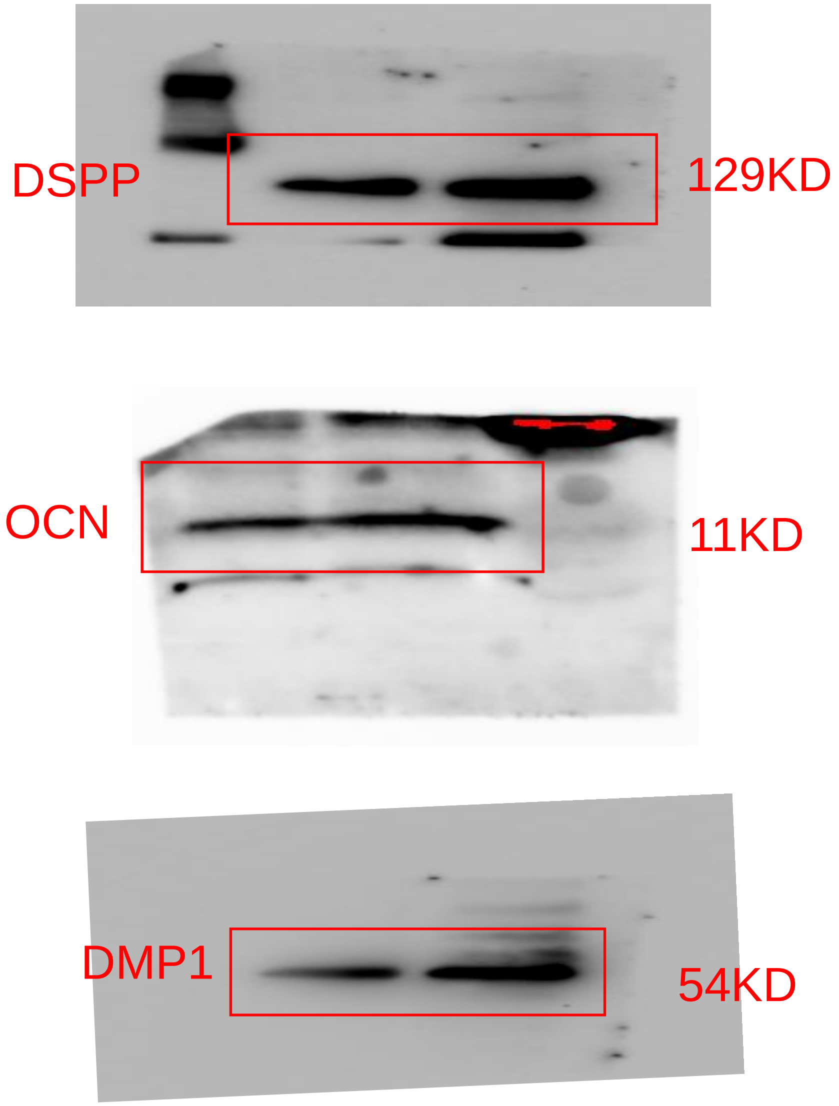

# Fig.3e

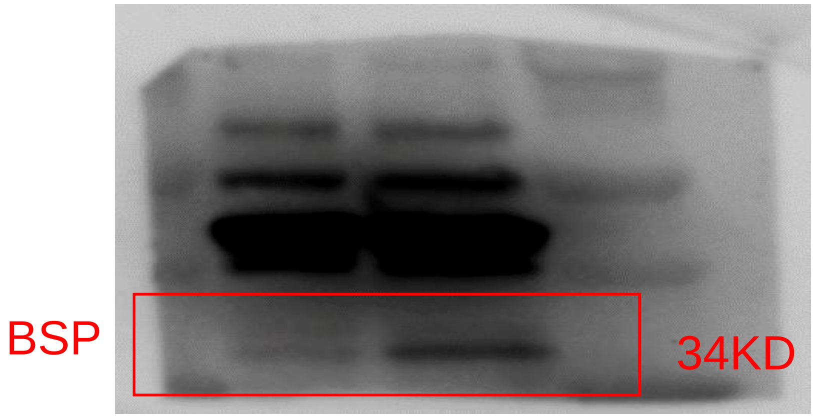


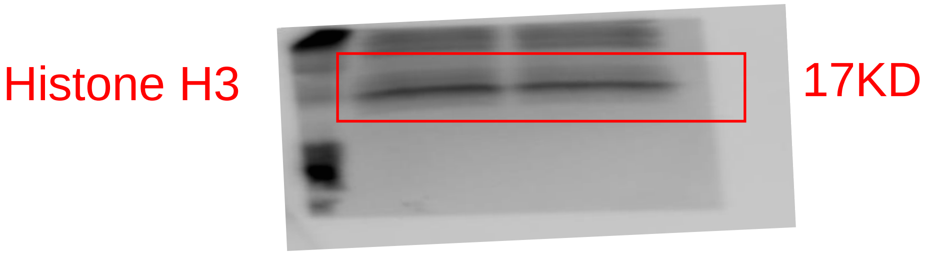

# Fig.3i

DMP1

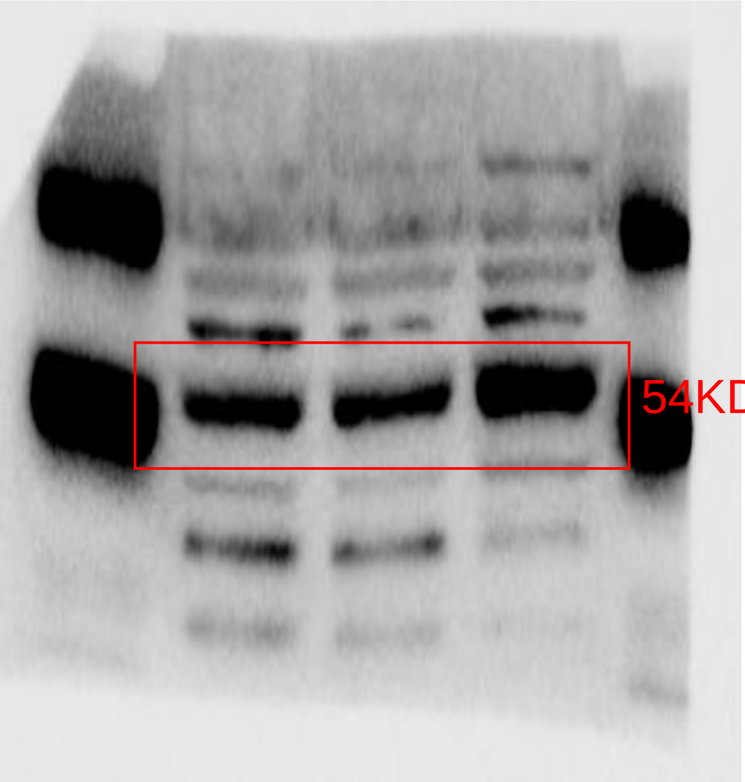

54KD

Histone H3

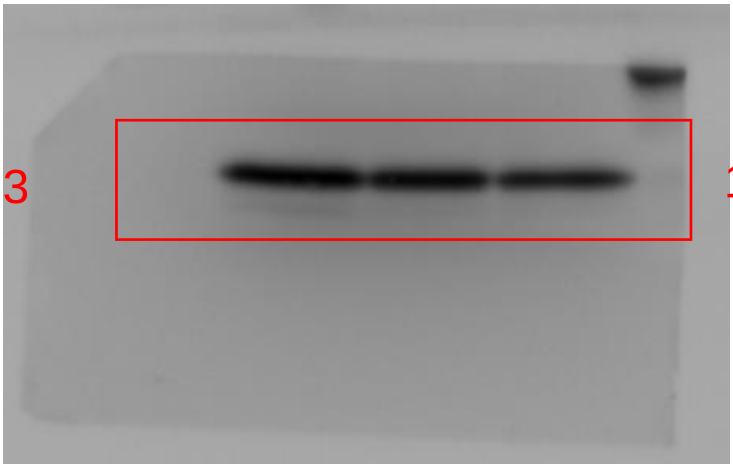

17KD

# Fig.3i

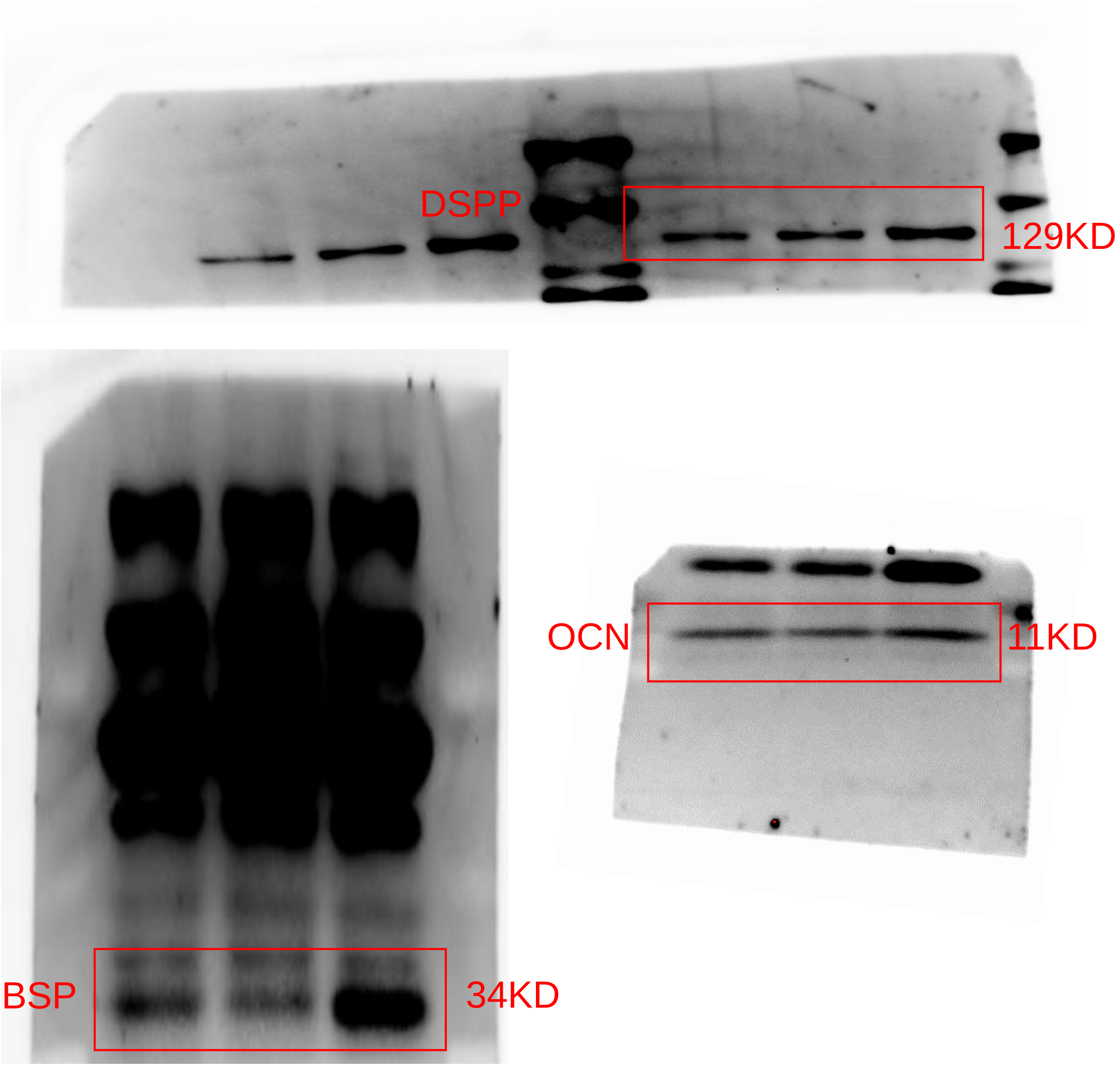

# Fig.5a

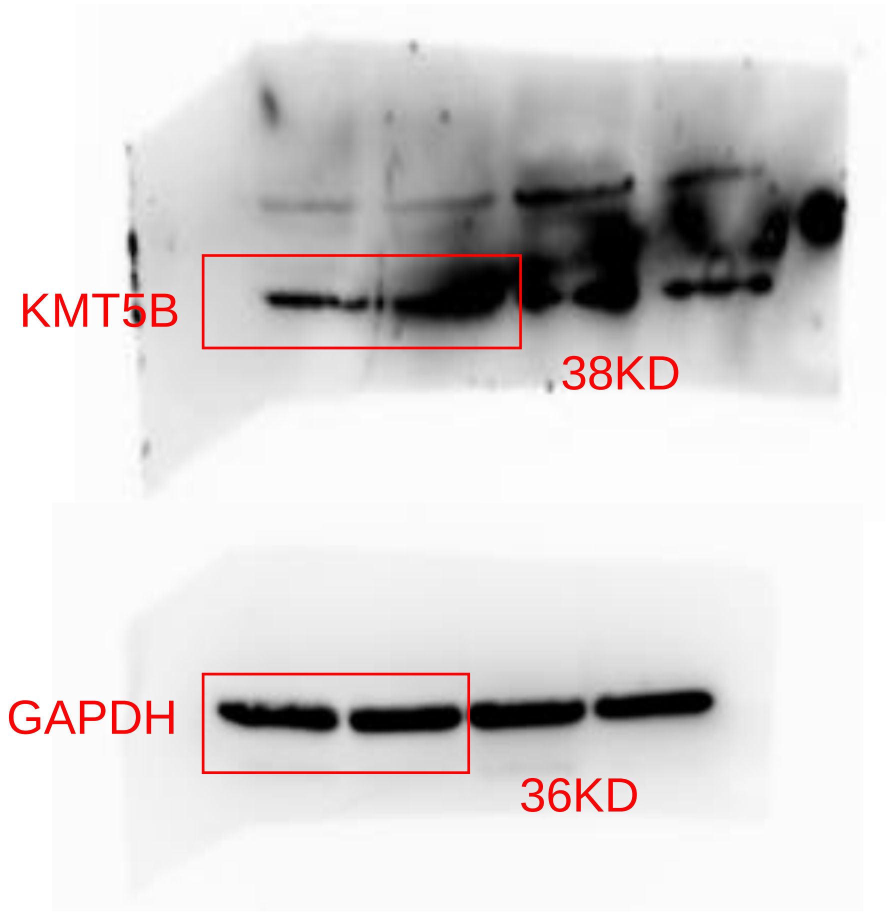

Fig.5e

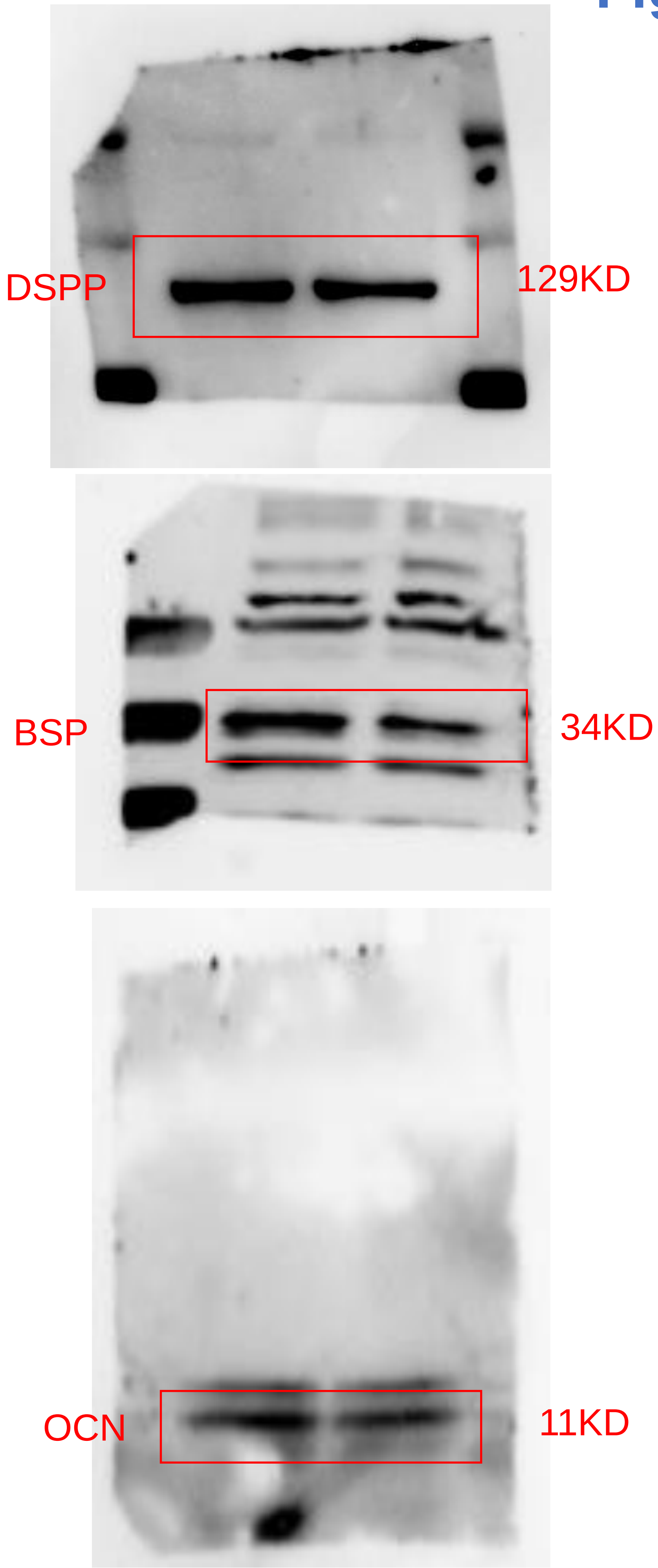

# Fig.5e

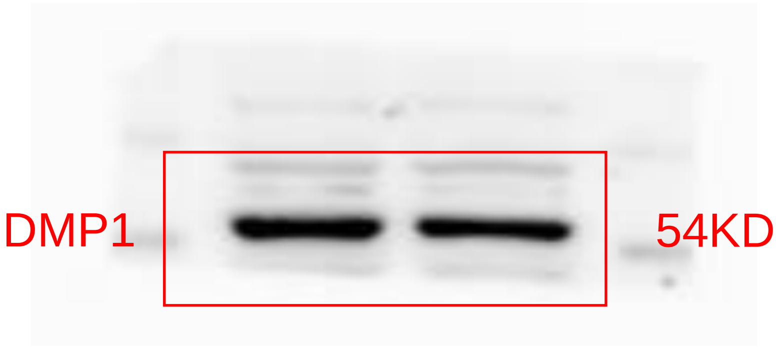


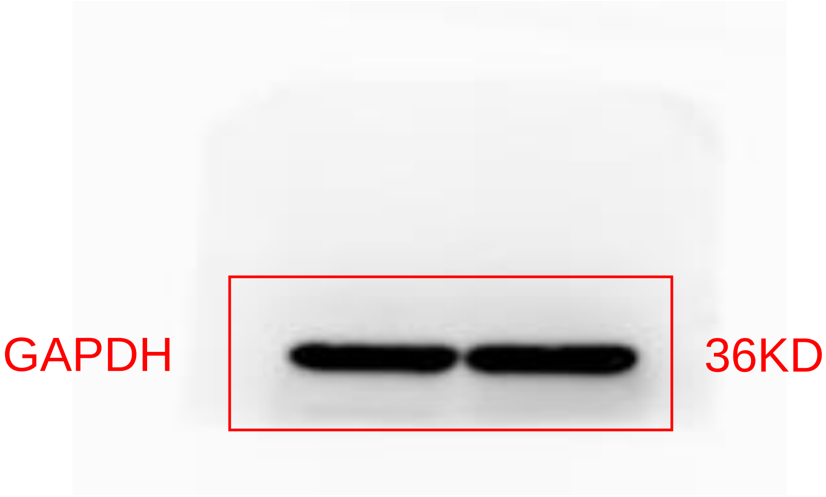

## Fig.6a

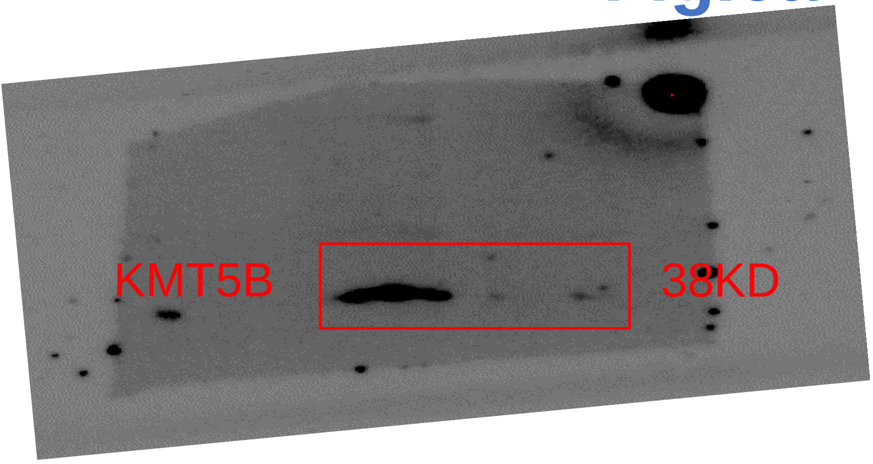


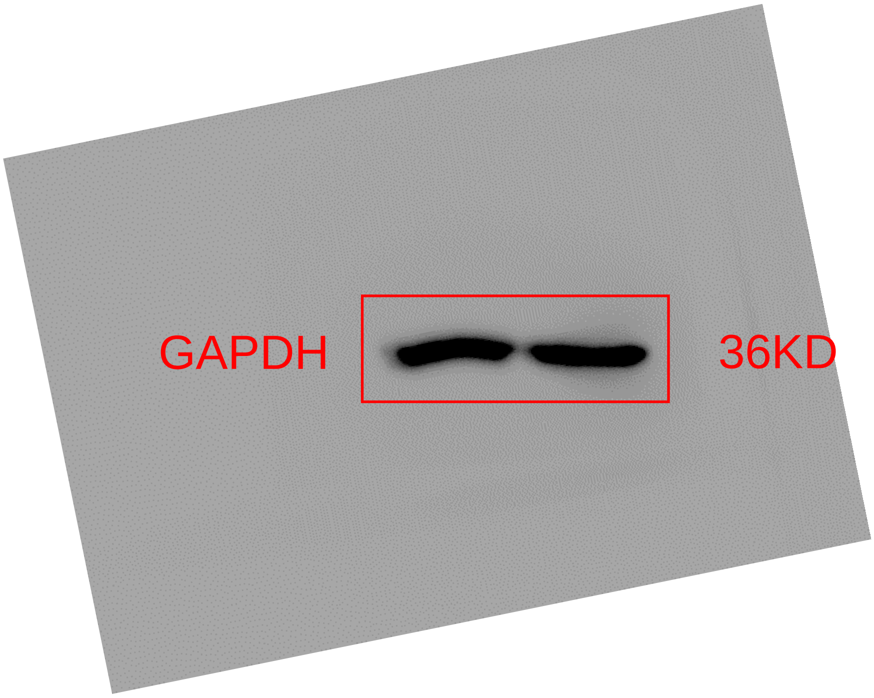

# Fig.6e

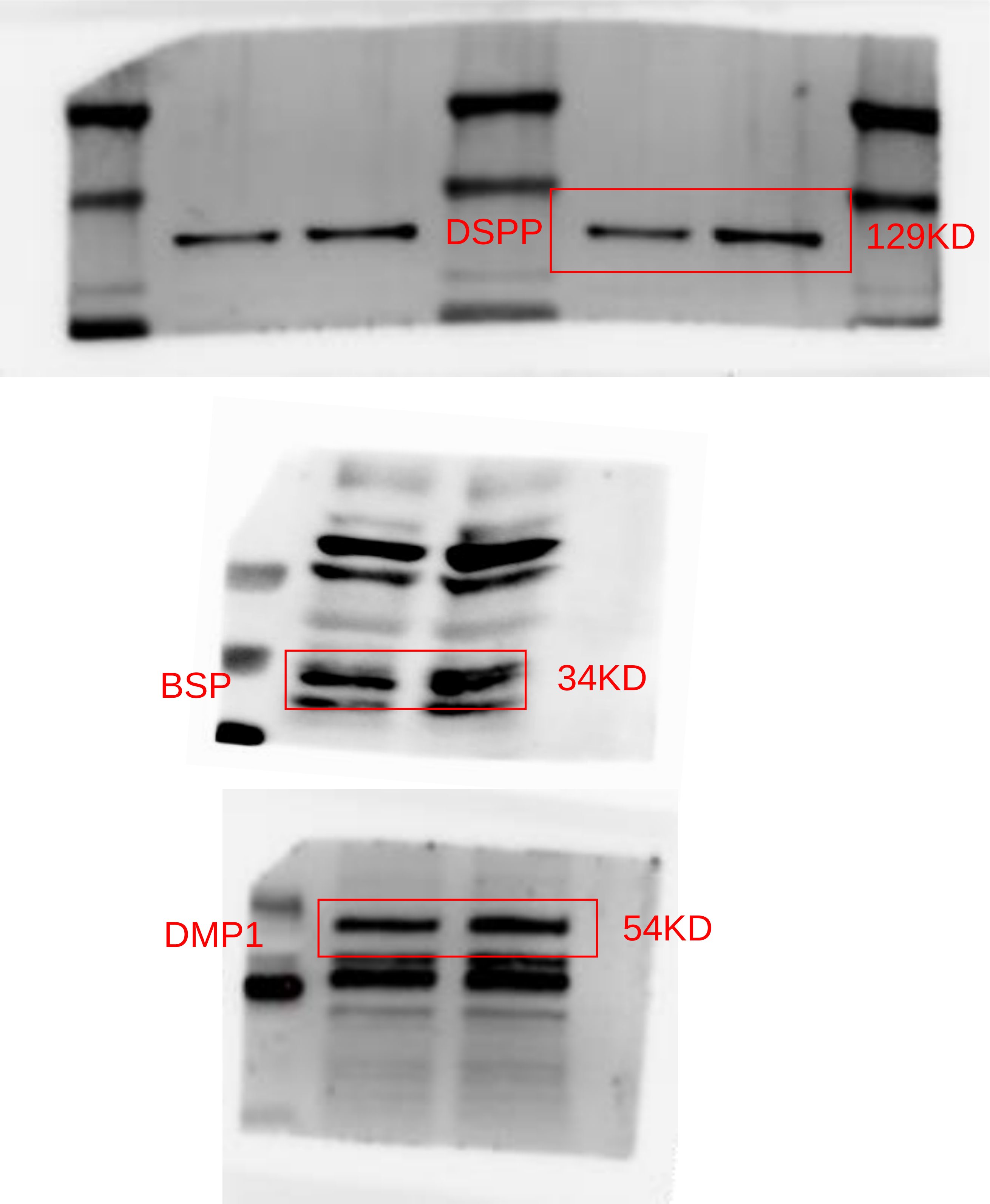

# Fig.6e

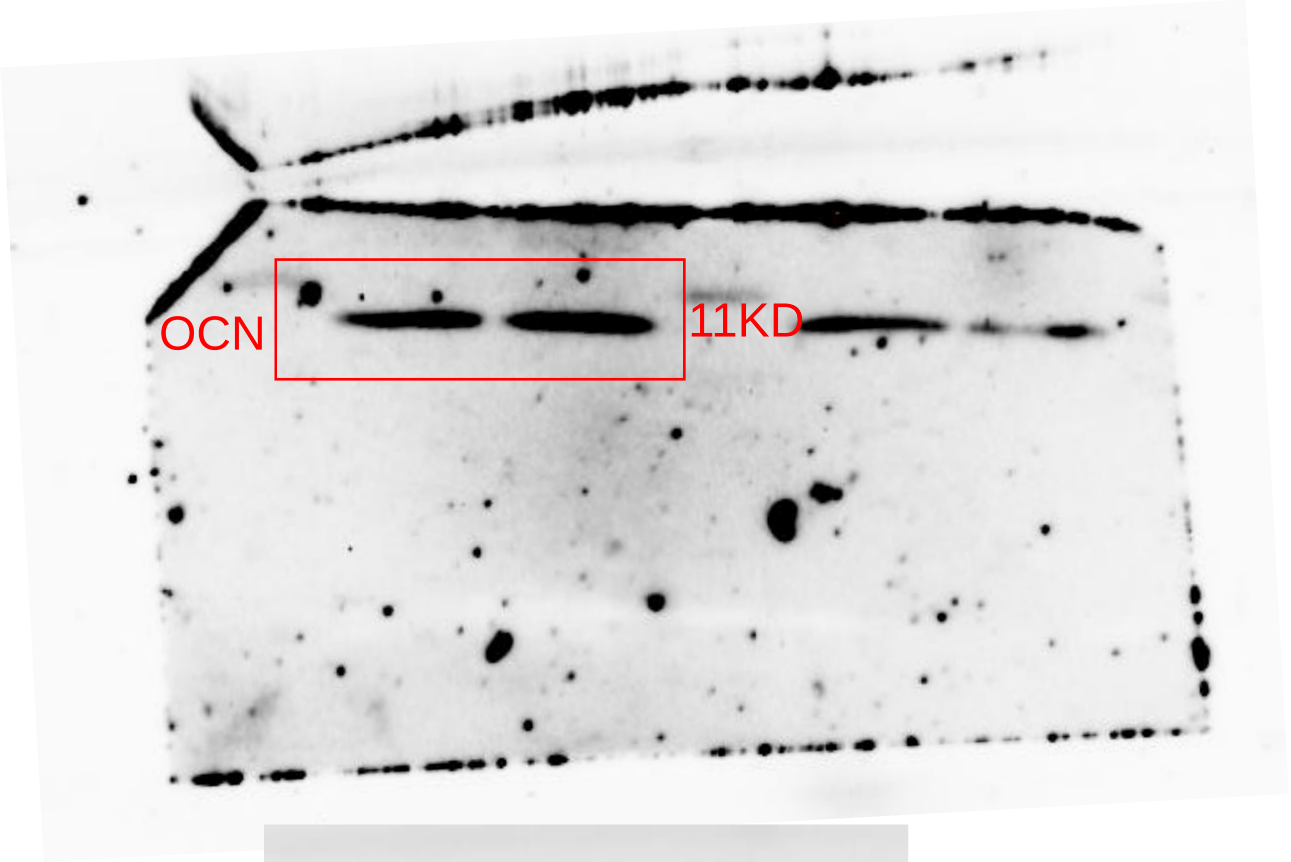


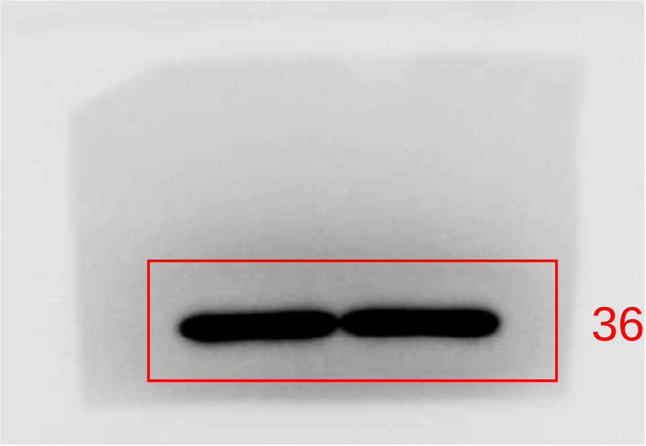

# Fig.6i

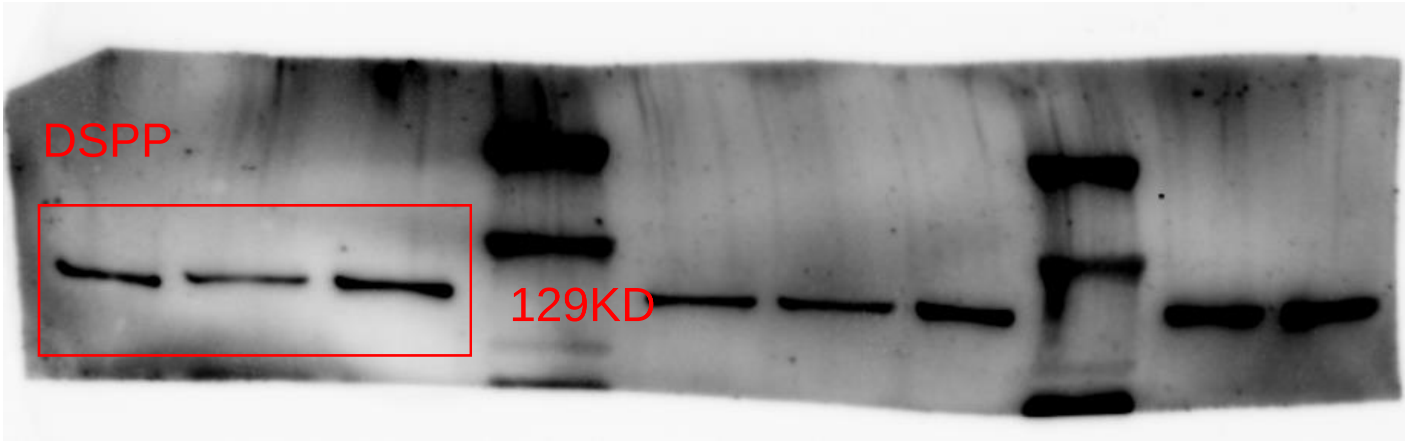


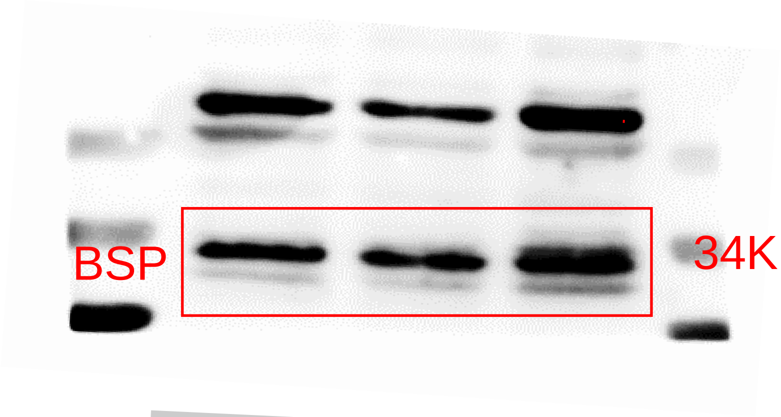


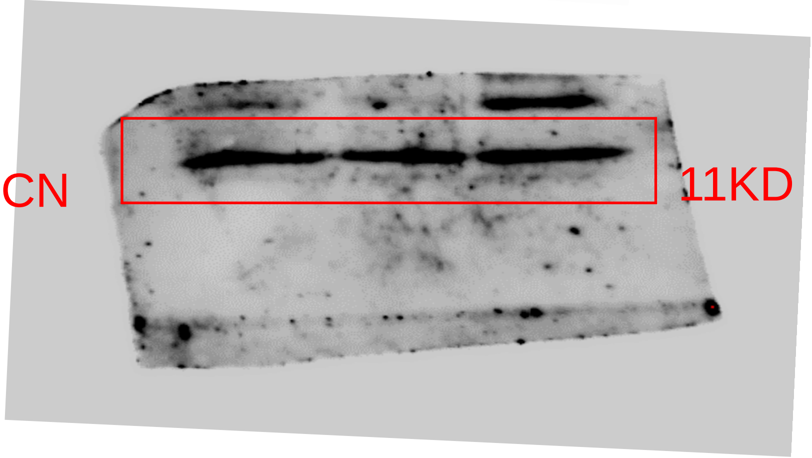

# Fig.6i

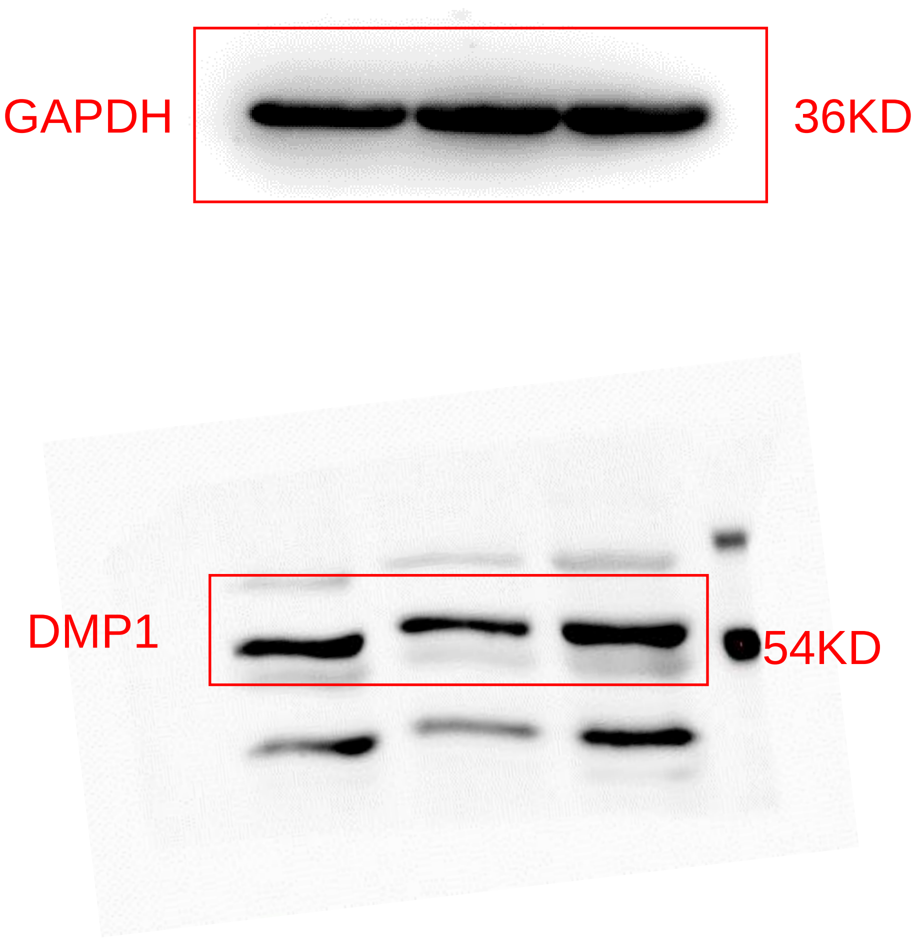

Supplement: Supplementary file 2 — western gels [file 41368_2021_148_MOESM2_ESM.pdf]
